# Ketamine disrupts naturalistic coding of working memory in primate lateral prefrontal cortex networks

**DOI:** 10.1038/s41380-021-01082-5

**Published:** 2021-05-12

**Authors:** Megan Roussy, Rogelio Luna, Lyndon Duong, Benjamin Corrigan, Roberto A. Gulli, Ramon Nogueira, Rubén Moreno-Bote, Adam J. Sachs, Lena Palaniyappan, Julio C. Martinez-Trujillo

**Affiliations:** 1 Department of Physiology and Pharmacology, University of Western Ontario, London, ON Canada; 2 Robarts Research Institute, University of Western Ontario, London, ON Canada; 3 Department of Psychiatry, University of Western Ontario, London, ON Canada; 4 Brain and Mind Institute, the University of Western Ontario, London, ON Canada; 5 Center for Neural Science, New York University, New York, NY USA; 6 Zuckerman Mind Brain Behavior Institute, Columbia University, New York, NY USA; 7 Center for Theoretical Neuroscience, Columbia University, New York, NY USA; 8 Center for Brain and Cognition, and Department of Information and Communication Technologies, Universitat Pompeu Fabra, Barcelona, Spain; 9 Serra Húnter Fellow Programme, Universitat Pompeu Fabra, Barcelona, Spain; 10 The Ottawa Hospital, University of Ottawa, Ottawa, ON Canada; 11 Lawson Health Research Institute, London, ON Canada

**Keywords:** Physiology, Neuroscience, Schizophrenia

## Abstract

Ketamine is a dissociative anesthetic drug, which has more recently emerged as a rapid-acting antidepressant. When acutely administered at subanesthetic doses, ketamine causes cognitive deficits like those observed in patients with schizophrenia, including impaired working memory. Although these effects have been linked to ketamine’s action as an N-methyl-D-aspartate receptor antagonist, it is unclear how synaptic alterations translate into changes in brain microcircuit function that ultimately influence cognition. Here, we administered ketamine to rhesus monkeys during a spatial working memory task set in a naturalistic virtual environment. Ketamine induced transient working memory deficits while sparing perceptual and motor skills. Working memory deficits were accompanied by decreased responses of fast spiking inhibitory interneurons and increased responses of broad spiking excitatory neurons in the lateral prefrontal cortex. This translated into a decrease in neuronal tuning and information encoded by neuronal populations about remembered locations. Our results demonstrate that ketamine differentially affects neuronal types in the neocortex; thus, it perturbs the excitation inhibition balance within prefrontal microcircuits and ultimately leads to selective working memory deficits.

## Introduction

Ketamine was developed as a dissociative anesthetic but more recently, at subanesthetic doses, it is used in medical practice as a rapid action antidepressant. It is additionally used as a recreational drug [1–5]. Through its action as an N-methyl-D-aspartate receptor (NMDAR) antagonist, it has been long known to induce a trance-like state providing pain relief, sedation, and memory loss [1, 5, 6]. Ketamine is also observed to induce negative, positive, and cognitive symptoms of schizophrenia [6–9]. Despite its widely observed effects, how ketamine induced blockage of NMDARs in individual synapses translate to cognitive and behavioral changes is still unclear.

For the particular case of ketamine induced cognitive deficits, some studies have hypothesized that ketamine decreases the stability of mental representations maintained by the primate lateral prefrontal cortex (LPFC) [10, 11]. Neuronal populations in the LPFC are thought to encode mental representations that are dissociable from sensory and motor signals and are therefore essential to processes like working memory (WM). However, because this part of the brain appears de novo in anthropoid primates and has a unique architecture relative to other phylogenetically older areas such as the medial prefrontal cortex, this hypothesis has been difficult to test in commonly used animal models, including rodents [12]. Illuminating how ketamine affects the function of primate lateral prefrontal microcircuits could explain its effects on human cognition as well as provide cautionary guidelines for its use in medical practice or as a recreational drug.

One prominent cognitive function that is impaired by ketamine is WM: the ability to temporarily hold and manipulate information relevant to a task [13]. This function is widely supported to depend on the activity of PFC neurons [14–20]. Previous studies have reported that NMDAR blockade by ketamine modulates single neuron activity within the LPFC during WM, leading to reduced neuronal tuning. [10, 21]. However, these studies have employed behavioral tasks involving simple visual displays relative to the complexity of natural environments and have strictly controlled for eye movements. This contrasts real-life settings, when WM representations must be held during dynamic viewing of natural scenes through saccades. Currently, it remains unknown whether neuronal population in LPFC can support WM function in ethologically valid settings and whether ketamine has any effect on WM function and brain microcircuit dynamics in these conditions. Here, we aimed to clarify this issue.

**Fig. 1:**
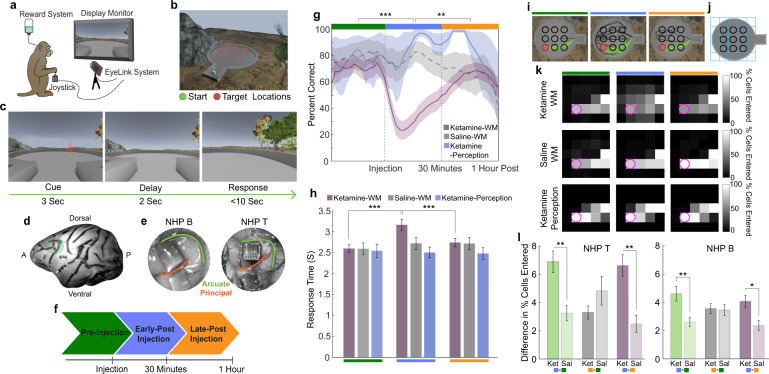
Virtual working memory task and behavioral performance. **a** Illustration of experimental setup. **b** Overhead view of task arena in virtual environment. **c** Trial epoch timeline. **d** Depiction of Utah array locations. **e** Surgical images of Utah arrays in LPFC. **f** Injection period timeline in which the pre-injection period refers to trials occurring before the time of injection, early post-injection period refers to injection time to 30 min post injection, and late post-injection period refers to 30 min post to 1 h post-injection time. Data from pre-injection period represented by green, early post-injection period by blue, and late post-injection period by orange. **g** Average percent of correct trials for ketamine-WM sessions (pink), saline-WM sessions (gray), and ketamine-perception sessions (blue). **h** Average response time for correct trials for all session types. **i** Trajectories to example target location (red) in one ketamine-WM session for correct (green) and incorrect (black) trials. **j** Task arena divided into 5 × 5 grid. **k** Percent of trials in which each cell of the arena is entered for example target location (pink) averaged over sessions. **l** Average difference (increase) in percent of trials in which cells are entered between injection periods (green = early post-injection – pre-injection; gray = late post-injection – pre-injection; purple = early post-injection – late post-injection) compared between ketamine-WM and saline-WM sessions. All error bars are SEM. *<0.05, **<0.01, ***<0.001.

We used a virtual reality engine to build a virtual arena featuring a naturalistic visual scene. We trained two rhesus monkeys (*Macaca mulatta*) on a visuospatial WM task that took place in this arena (Fig. 1a, b). As during natural behavior, animals were permitted free visual exploration (unconstrained eye movements), as well as free spatial navigation using a joystick. During task trials, a target was presented for 3 s at 1 of 9 locations in the arena. The target then disappeared during a 2 s delay epoch. During the target and delay epoch, navigation was disabled. Subsequently, navigation was enabled, and animals were required to virtually approach the target location within 10 s to obtain a juice reward (Fig. 1c). We recorded neuronal activity during this task using 96-channel microelectrode arrays (Utah Arrays). Two arrays were implanted in each animal in the left LPFC, one on each side of the principal sulcus (Fig. 1d, e) [22].

In order to block NMDARs, we administered ketamine intramuscularly. NMDARs are evidenced to be critically involved in balancing prefrontal circuit interactions between pyramidal cells and inhibitory interneurons that are crucial for WM processing [10, 23–25]. Ketamine is reported to impair WM performance through primarily blocking NMDARs, which are highly expressed in the human prefrontal cortex [6, 8, 10, 25, 26]. Local administration of NMDAR antagonists into the primate LPFC is also sufficient to perturb WM signals [10]. Accordingly, it is reasonable to assume that low doses of systemically administered ketamine would produce the greatest effect on prefrontal neuronal activity [8, 27].

We recorded neuronal responses during the task in three blocks of trials, which were defined relative to the injection time. Blocks were chosen based on ketamine’s intramuscular post-injection peak plasma point (5 min) and observed time of action (3–30 min) [28]. Trial blocks were therefore defined as: before subanesthetic ketamine (0.25–0.8 mg/kg) or saline injection (pre-injection period), 30 min post injection (early post-injection period), and 30 min post injection to 1 h post injection (late post-injection period) (Fig. 1f). In some sessions, we used a control task in which targets remained on screen for the duration of the trial (ketamine-perception variant). Here, the animals did not have to remember the target location; therefore, WM was not required to complete the trials. This control variant of the task allowed us to separate the effect of ketamine on WM function from potential effects on processes like perception and movement.

We hypothesized that neuronal populations in LPFC would robustly encode WM information in our naturalistic WM task. We further hypothesized that ketamine would selectively impair WM performance by disrupting the tuning of single neurons as well as the amount of information encoded by neuronal populations about remembered locations.

## Results

### Ketamine impairs behavioral performance in a naturalistic working memory task

The following results are divided based on the three injection periods defined by their temporal relationship to the injection time: pre-injection (prior to injection), early-post injection (up to 30 min post injection), and late-post injection (30 min post injection to 1 h post injection). Both animals performed significantly above chance (~11%, nine locations) on all task variants before ketamine injections (pre-injection period, *p* < 0.001), indicating proficiency in the task. Performance differed significantly between injection periods (Two-way ANOVA, *F*(2,69) = 4.3, *p* = 0.017) and between saline and ketamine sessions (Two-way ANOVA, *F*(1,69) = 9.57, *p* = 0.003). In ketamine-WM sessions, performance decreased significantly during the early post-injection period compared to the pre-injection period (Two-way ANOVA, post hoc, *p* < 0.0001), to subsequently recover during the late post-injection period compared to the early post-injection period (Two-way ANOVA, post hoc, *p* = 0.002). Performance did not significantly change between injection periods in saline-WM sessions (Two-way ANOVA, post hoc, pre-injection and early post-injection, *p* = 0.999). Importantly, ketamine injections did not significantly alter performance between injection periods in perception sessions (ANOVA, *F*(2,6) = 0.25, *p* = 0.786), indicating that the ketamine induced performance deficit was specific to the WM task (Fig. 1g) (see data per animal in Fig. S1 a, b).

Navigation time to the remembered target location also significantly varied between injection periods (ANOVA, *F*(2,250) = 16.81, *p* < 0.0001). Navigation time increased significantly after ketamine injection compared to the pre-injection period (ANOVA, post hoc, *p* < 0.0001) and decreased in the late post-injection period compared to the early post-injection period (ANOVA, post hoc, *p* < 0.0001). No significant changes were found between injection periods in saline-WM (ANOVA, *F*(2,108) = 1.71, *p* = 0.186) or ketamine-perception sessions (ANOVA, *F*(2,60) = 0.22, *p* = 0.800) (Fig. 1h; see data per animal in Fig. S1 c, d).

Trajectories to remembered targets also became more dispersed after ketamine injections in the early post-injection period compared to the pre-injection period (Fig. 1i). To quantify this observation, we divided the task environment into a 5 × 5 grid creating 25 regional cells (see Fig. 1j) and calculated the percent of trials in which each cell was entered during navigation to a target location (Fig. 1k). The difference in the percent of trials in which cells were entered between pre and post-injection periods in ketamine-WM and saline-WM sessions was then calculated. In ketamine-WM sessions, more cells were visited in more trials in the early post-injection compared to the pre-injection period relative to saline-WM sessions (Two-way ANOVA, post hoc, animal T, *p* = 0.002; animal B, *p* = 0.004). Fewer cells were visited in the late post-injection period compared to the early post-injection period in ketamine-WM sessions compared to saline-WM sessions (Two-way ANOVA, post hoc, animal T, *p* = 0.001; animal B, *p* = 0.044) (Fig. 1l). We observed less dispersion of the trajectories in the post-injection period relative to the pre-injection period during ketamine perception sessions compared to ketamine WM sessions (Fig. 1k last row, Two-way ANOVA, post hoc, *p* < 0.0001). These results indicate that ketamine selectively impaired the animals’ ability to maintain the location of the target in WM.

### Ketamine decreases tuning of single neurons for remembered locations

**Fig. 2:**
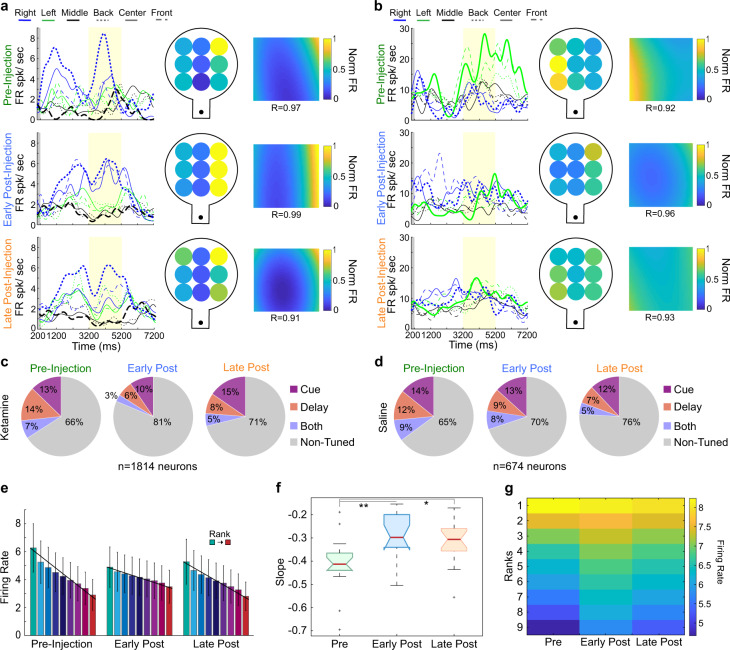
Ketamine decreases tuning of single neurons for remembered locations. **a** Firing rate of an example neuron for a ketamine-WM session. On the left, spike density functions (SDFs) over the pre-cue interval (gray column), cue, delay (yellow), and response epochs. Preferred locations and least-preferred locations are bolded. Center, firing rates during the delay epoch for all target locations. Right, firing rates fitted to a polynomial plane. **b** Firing rate of a second example neuron during a ketamine-WM session. **c** Average proportion of tuned single units during the cue epoch (pink), delay epoch (orange), or during both (purple) for each injection period for ketamine-WM sessions. **d** Average proportion of tuned single units during each epoch for saline-WM sessions. **e** Example session indicating firing rate averaged over neurons for target locations ranked from preferred to least-preferred locations. Black lines represent slope. **f** Fitted slope for each injection period averaged over sessions. **g** Firing rate for each target location ranked and averaged over sessions for each injection period. All error bars are SEM. *<0.05, **<0.01, ***<0.001. Red center lines indicate median, the bottom and top edges of the box indicate the 25th and 75th percentiles. The whiskers extend to non-outlier data points (approximately within 2.7 std) and the outliers are plotted using ‘+’.

To investigate the neuronal correlates of the behaviors illustrated in Fig. 1, we recorded the activity of 2906 units (1814 single neurons and 1092 multiunits) during 17 ketamine-WM sessions (8 in animal T, 9 in animal B). We recorded an additional 1117 units (674 single units and 443 multiunits) during seven saline-WM sessions (3 in animal T, 4 in animal B). Single neurons exhibited spatial tuning for cued locations during the delay epoch in the pre-injection period (example neurons in Fig. 2a, b). We compared the proportion of tuned units between injection periods during ketamine-WM and saline-WM sessions. In ketamine sessions, the proportion of spatially tuned neurons significantly decreased in the early post-injection period compared to the pre-injection period (Chi-Square, *X*^2^ = 128.67, *p* < 0.0001) and significantly increased in the late post-injection period compared to the early-post injection period (Chi-Square, *X*^2^ = 126.52, *p* < 0.0001) (Fig. 2c; see data per animal in Fig. S2a, b). There were no significant differences in the proportion of tuned single neurons between pre-injection and early post-injection periods during saline-WM sessions (Chi-Square, *X*^2^ = 1.44, *p* = 0.231) (Fig. 2d).

We additionally analyzed tuning functions of single neurons by ranking their responses per target location during the delay epoch in the three injection periods. We computed the slope of a straight line fitted to the responses (Fig. 2e shows data pooled across neurons from one example session). Slope magnitude changed significantly between injection periods (Kruskal–Wallis, *H*(2,48) = 13.48, *p* = 0.001). The slopes significantly decreased in magnitude during the early post-injection period compared to the pre-injection period (Kruskal–Wallis, post hoc, *p* = 0.001) (Fig. 2f, g). This was not the case for the saline control sessions (Kruskal–Wallis, *H*(2,18) = 5.7, *p* = 0.058). These results demonstrate that single neurons in LPFC encode spatial WM signals in naturalistic conditions and that low doses of ketamine significantly impair single neuron tuning.

### Ketamine disrupts population decoding of remembered locations

**Fig. 3:**
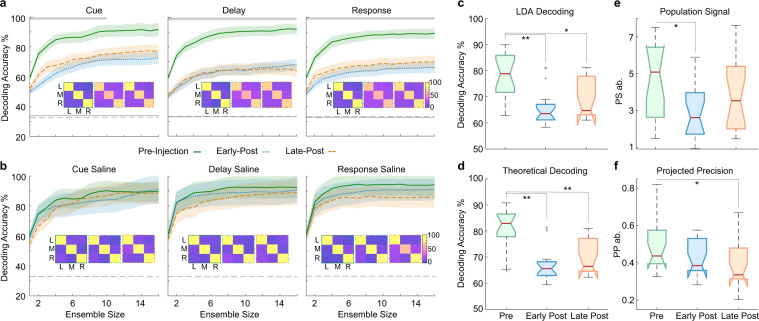
Neuronal population decoding of target locations. **a** Median decoding accuracy for ketamine-WM sessions for pre-injection (green), early post-injection (blue), and late post-injection periods (orange) for trial epochs. Chance performance is indicated by dashed gray line and shuffled results are indicated by solid gray line. Confusion matrices for each injection period indicate classifier performance for each target location. Gray bars near the top of the plot indicate ensemble sizes showing a significant reduction in decoding accuracy from pre-injection to early post-injection periods (Kruskal–Wallis, *p* < 0.05). **b** Same as (**a**), for saline-WM sessions. All error bars are SEM. **c** DPe over the delay epoch for ketamine-WM sessions. **d** DPt over the delay epoch for ketamine-WM sessions. **e** PS over the delay epoch for ketamine-WM sessions. **f** PP over the delay epoch for ketamine-WM sessions. Red center lines indicate median, the bottom and top edges of the box indicate the 25th and 75th percentiles. The whiskers extend to non-outlier data points (approximately within 2.7 std) and the outliers are plotted using ‘+’. *<0.05, **<0.01, ***<0.001.

Single neuron tuning is essential for information coding. However, the information encoded by a neuronal population also depends on the correlated activity of neurons and can only be accurately estimated by examining the activity of simultaneously recorded neurons [20, 29]. We used a linear classifier (Support Vector Machine, SVM) to predict from neuronal ensemble activity whether targets were presented on the left, right, or center of the virtual arena on a single trial basis. We pooled locations in order to reach a sufficient sample size (trials) to use cross-validation procedures. Decoding accuracy for different ensemble sizes was higher than chance (33%) in all analyzed experimental sessions (Fig. 3a, b and Fig. S3). Decoding accuracy decreased after ketamine injection between pre-injection and early post-injection periods (Fig. 3a), predominantly during the delay and response epochs (16 neuron ensemble, Kruskal–Wallis, post hoc: delay; *p* = 0.015, response; *p* = 0.023). The classifier made systematically more errors after ketamine injection. Similar results were observed when using only correct trials or decoding 9 target locations in sessions with sufficient sample sizes (Fig. S4). On the other hand, decoding accuracy remained stable between injection periods in saline-WM sessions (16 neuron ensemble, Kruskal–Wallis: delay; *H*(2,18) = 1.12, *p* = 0.571, response; *H*(2,18) = 1.36, *p* = 0.507) (Fig. 3b). These results indicate that LPFC neuronal ensembles encode spatial WM in naturalistic settings and that ketamine disrupts these ensemble codes.

### Ketamine decreases decoding by reducing the reliability of the population signal

In order to further understand how ketamine impacts LPFC population codes, we explored the statistical properties underlying information coding: the population signal (PS) and the projected precision (PP) [29]. PS reflects the modulation of the population response across target locations (i.e., the vector of differences between population responses to different target locations). Therefore, a decrease in tuning for target location would produce a decrease in PS. PP reflects the correlated variability of neuronal responses projected onto the PS vector (i.e., projection of the covariance matrix inverse on the direction of the PS vector). It is important to distinguish between PP and average noise correlations. Variations in noise correlations alone cannot predict changes in information coding [29]. It is possible that the effect of ketamine on neuronal coding occurs through modulation of either PS (neuronal tuning), PP (relationship between population noise correlation structure and PS direction) or both.

To investigate how ketamine decreased the ability of neuronal populations to discriminate between remembered locations, we used binary classes (left vs. right locations) and ensembles of 3 neurons, which allowed for large enough sample sizes to reliably compute PS and PP [29]. We selected random ensembles providing the highest empirical decoding accuracies for the remembered location (within the top 75th percentile calculated using Linear Discriminant Analysis, LDA). We first compared decoding accuracy results using empirical decoding (LDA) and theoretical decoding (using PP and PS) and demonstrated no significant differences (Kruskal–Wallis, *H*(1,100) = 1.87, *p* = 0.171) (Fig. S5a, b). This control was required to show that our theoretical decoding method employing PP and PS accurately estimated information content of neuronal ensembles.

As shown in Fig. 3c and Fig. 3d, there was a significant change in empirical decoding accuracy (DPe; Kruskal–Wallis, *H*(2,48) = 13.37, *p* = 0.001) and theoretical decoding accuracy (DPt; Kruskal–Wallis, *H*(2,48) = 17.96, *p* = 0.0001) between injection periods. There was an increase in decoding accuracy between the pre and early post-injection periods for empirical decoding (DPe; Kruskal–Wallis, post hoc, *p* = 0.001) and theoretical decoding (DPt; Kruskal–Wallis, post hoc, *p* = 0.0002) and an increase during the late post-injection compared to the early post-injection period (for data per animal see Fig. S5c-f). Decoding accuracy in saline-WM sessions did not significantly change between injection periods (DPe, Kruskal–Wallis, *H*(2,18) = 2.43, *p* = 0.297; DPt, Kruskal–Wallis, *H*(2,18) = 3.95, *p* = 0.139) (see Fig. S5g, h).

Notably, PS significantly differed between injection periods in ketamine-WM sessions (Kruskal–Wallis, *H*(2,48) = 8.13, *p* = 0.017). PS decreased after ketamine injection (early post-injection) compared to the pre-injection period (Kruskal–Wallis, post hoc, *p* = 0.012) and increased during the late post-injection compared to the early post-injection period (Fig. 3e). PP showed a small decrease between injection periods (Kruskal–Wallis*, H*(2,48) = 8.2, *p* = 0.017). There was a non-significant decrease in the early post-injection period compared to the pre-injection period (Kruskal–Wallis, post hoc, *p* = 0.380) (Fig. 3f) (for data per animal see Fig. S5i-l). The PP decrease became significant during the late post-injection relative to the pre-injection period (Kruskal–Wallis, post hoc, *p* = 0.012). The latter result may suggest that ketamine induced slow changes in correlated variability or its projection onto the PS vector, which outlasted changes in neuronal tuning. Overall, these results indicate that the observed early changes in information decoded from populations of neurons after ketamine injection is primarily due to changes in PS, a consequence of changes in individual neuron tuning.

### Ketamine has differential effects on excitatory and inhibitory cell types

**Fig. 4:**
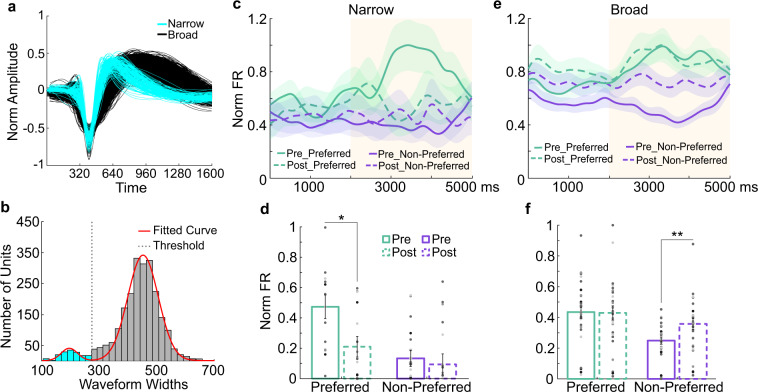
Cell type specific effects of ketamine on working memory signals. **a** Waveforms of narrow and broad spiking neurons. **b** Distribution of waveform widths (microseconds) fitted with a 2-Gaussian model. Boundary line between narrow and broad spiking neurons is at the intersection point between Gaussians (275, dotted line). Gaussian at the lower width boundary indicates narrow spiking neurons (blue) and the upper boundary indicates broad spiking neurons (dark gray). **c** Normalized average population SDFs for cue and delay (yellow) epochs for delay tuned narrow spiking neurons. **d** Median population SDF for narrow spiking neurons over the delay epoch. Data points represent value per electrode array for each session. **e** Normalized average population SDF for cue and delay epochs (yellow) for delay tuned broad spiking neurons. **f** Median population SDF for broad spiking neurons over the delay epoch. All error bars are SEM. *<0.05, **<0.01, ***<0.001.

Ketamine induces a variety of effects on individual neurons [10, 30]. A loss of neuronal tuning may result from neurons increasing their response to least-preferred locations (see example neuron Fig. 2a) or decreasing their response to preferred locations (see example neuron Fig. 2b). One possible explanation for this heterogeneity is that different cell types (e.g., excitatory pyramidal cells and inhibitory interneurons) may be differentially affected by ketamine. To test this hypothesis, we divided neurons that were tuned during the delay epoch into narrow and broad spiking (BS) based on waveform peak-to-trough duration (width) (Fig. 4a, b). In mouse neocortex, BS neurons are largely putative pyramidal cells or in a smaller proportion, vasointestinal peptide expressing (VIP) neurons. On the other hand, narrow spiking neurons are largely parvalbumin (PV) expressing, or in a smaller proportion, somatostatin expressing inhibitory interneurons [31].

We then calculated the firing rates for each neuron’s preferred and least-preferred target locations during the pre-injection and post-injection periods. After ketamine injection (early post-injection), narrow spiking neurons showed a loss of tuning during the delay epoch due to a decrease in firing for their preferred locations compared to the pre-injection period (Wilcoxon Rank-sum, *p* = 0.049) with no significant change for their least-preferred locations (Wilcoxon Rank-sum, *p* = 0.546) (Fig. 4c, d). In contrast, BS neurons showed a loss of tuning due to a significant increase in firing for their least-preferred locations compared to the pre-injection period (Wilcoxon Rank-sum, *p* = 0.006) with no significant change for their preferred locations (Wilcoxon Rank-sum, *p* = 0.649) (Fig. 4e, f). Such changes were not observed during saline-WM sessions (Fig. S6a, b; data per animal in Fig. S6e-l). We also conducted separate analyses of PS in narrow and BS single neurons and found a loss of PS and a resultant decrease in DPt in both populations (see Fig. S7).

Considering that our populations of NS and BS neurons are dominated by PV and pyramidal cells respectively, our findings align with a proposed pathophysiological mechanism for WM dysfunction: reduced NMDAR conductance on inhibitory PV interneurons, amounting to generalized disinhibition of pyramidal cells and resultant loss of tuning [11, 30]. Indeed, ketamine has high affinity for GluN2B NMDAR subunits which are expressed in PV interneurons [2, 32]. Loss of pyramidal cell tuning reduces the spatial specificity of WM representations, the PS, and encoded information by a population of neurons regarding remembered target location.

### Ketamine did not affect gaze behavior

**Fig. 5:**
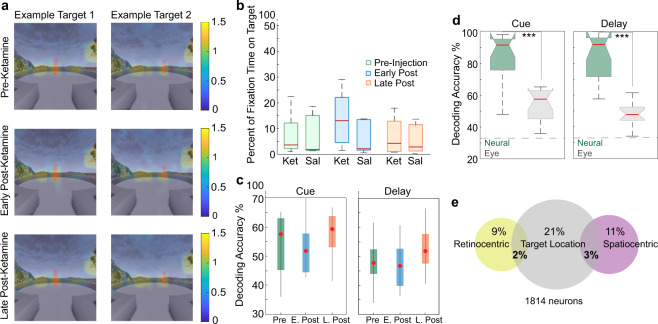
Effect of ketamine on gaze behavior. **a** Heat maps indicating eye fixation locations for two example target locations during the different injection periods. **b** Proportion of fixation time falling on the target location compared to all fixation time during the delay epoch for ketamine and saline sessions. **c** Comparison of decoding accuracy for target locations using eye fixation position between pre, early, and late- post ketamine-injection periods for the cue and delay epochs. **d** Comparison between decoding target location accuracy using neuronal ensemble activity (green) and eye fixation position on screen (gray) during the ketamine pre-injection period for the cue and delay epochs. **e** Proportion of single units tuned for target location during the delay epoch and tuned for saccade position in retinocentric or spatiocentric reference frames. Red center lines indicate median, the bottom and top edges of the box indicate the 25th and 75th percentiles. The whiskers extend to non-outlier data points (approximately within 2.7 std) and the outliers are plotted using ‘+’. *<0.05, **<0.01, ***<0.001.

A proportion of neurons in the LPFC encode signals related to gaze [33]. Since gaze was unconstrained in our task, it is possible that the coding of remembered locations predominantly reflect systematic biases in eye position signals. To explore this possibility, we first determined whether animals showed biases in eye position toward the target location (see example target locations in Fig. 5a). We calculated the duration in which the position of eye fixation was directed to the target location during the delay epoch divided by total time in which animals were fixating during the delay. We found that only 3.6% of fixation time during the delay epoch was spent looking at the target location in the pre-injection period. There were no significant differences between injection periods or between saline and ketamine sessions (Two-way ANOVA, drug, *F*(1,69) = 1.73, *p* = 0.193, injection period, *F*(2,69) = 1.42, *p* = 0.248, interaction, *F*(2,69) = 1.35, *p* = 0.267 (Fig. 5b).

As an additional measure, we used a linear classifier to predict target location from the position of eye fixations on the screen. We divided the screen into 16 cells and calculated the number of fixations falling within each cell. During the pre-injection period, the accuracy for decoding remembered locations from fixations was significantly higher than chance, indicating a target specific gaze bias (cue: *t*-test, *T*(15) = *8.38, p* < 0.0001, delay: *T*(15) = 8.53, *p* < 0.0001; Fig. 5c, d). Such a bias was less pronounced during the delay relative to the cue epoch (Wilcoxon Rank-sum, *p* = 0.002; Fig. 5c). However, decoding accuracy for remembered locations from eye position was significantly lower than decoding accuracy of a classifier that uses neuronal firing rate and the same number of features (*n* = 16) (Kruskal–Wallis, cue: *H*(1,30) = 14.78, *p* = 0.0001; delay: *H*(1,30) = 22.91, *p* < 0.0001; Fig. 5d). Together, this data suggests that biases in eye position signals are not sufficient to account for the amount of information encoded by the population activity regarding target location.

After ketamine injections (early post-injection), decoding accuracy for remembered locations from eye position remained stable compared to the pre-injection period, Kruskal–Wallis; cue, *H*(2,45) = 4.01, *p* = 0.135, delay, *H*(2,45) = 4.59, *p* = 0.101; Fig. 5c). On the other hand, decoding accuracy for remembered locations from neuronal activity significantly decreased after ketamine injection (delay: Kruskal–Wallis, *H*(2,45) = 11.26, *p* = 0.004, post hoc, *p* = 0.015) (Fig. 3a). These results indicate that biases in eye position cannot account for the effects of ketamine on decoding of target locations from neuronal activity and suggest a dissociation between eye position and WM signals within LPFC microcircuits.

Finally, we calculated the proportion of single units tuned for eye position in both retinocentric and spatiocentric reference frames using Kruskal–Wallis analysis of variance. Using the retinocentric reference frame, saccade landing position was determined relative to the starting point of the saccade, independent from the landing location on the screen. In a spatiocentric reference frame, saccades were characterized according to their landing position on the screen, independent from the saccade starting position [34]. During the delay epoch, 9% of the neurons showed tuning for saccades in a retinocentric reference frame and 11% in a spatiocentric frame. However, only 2% of single units were tuned for both target location and saccades in the retinocentric frame and 3% of single units were tuned both for target location and saccades in spatiocentric frame (Fig. 5e). These results indicate that only a small number of neurons were tuned for eye position, and from those, only a small fraction were tuned for WM representations of target location. These results further argue against eye position related activity as the explanation for the coding of target position during the delay epoch.

## Discussion

We used multielectrode arrays to simultaneously record the responses of single units in the macaque LPFC (pre/periarcuate areas 8 A/46) [22] before and after administering subanesthetic doses of ketamine. We report three major findings: (1) ketamine selectively perturbs WM representations of targets in a naturalistic spatial WM task, (2) this effect is mediated by reduced spatial tuning of individual neurons leading to a loss of encoded information regarding target location at a neuronal population level, (3) ketamine induced changes in neuronal tuning were due to different effects on narrow and BS neurons; response decrease in the former and response increase in the latter.

Our study shows that macaque LPFC neurons encode WM representations during naturalistic tasks, regardless of potential interference by sensory and motor signals generated during natural behavior. Thus, the LPFC differs from areas such as the posterior parietal cortex where WM representations are perturbed by visual distractors [15] and the frontal eye fields where shifts in gaze disrupt WM signals [35]. Indeed, previous studies exploring the effects of ketamine on prefrontal neuronal activity while maintaining strict control of eye position show similar results to ours. Using the traditional spatial WM oculomotor delayed response task, which controls for eye position, Wang et al. [10] found that persistent activity in a small sample of delay cells was abolished and that spatial tuning was reduced after administration of specific NMDAR antagonists as well as systemic ketamine. In a rule-based WM task that restricted eye position, systemic ketamine decreased the rule signal during the delay epoch (i.e., differences in neuronal responses to prosaccade and anti-saccade trials). This result agrees with the loss of PS reported in our study [21].

The granular LPFC, an anthropoid primate specialization, may allow for the encoding of representations that are uniquely dissociated from distraction and action. This seems to differ from the rodent prefrontal cortex, where neurons primarily encode prospective information about movement plans [36]. Thus, the granular LPFC may have allowed expanding the mental world of primates; consequently, enhancing their adaptability to changing environments [12, 37]. However, it may have also brought about new vulnerabilities upon which particular types of mental diseases develop as well as susceptibility to certain drugs.

One may argue that a limitation of our study was that ketamine was administered systemically, and since we recorded from LPFC, we may have not been able to observe effects in other brain regions. This is possible; however, the observed effects of ketamine were specific to WM and resembled those of early lesion studies in the same region [18]. Moreover, local iontophoresis of NMDAR blocker, MK-801, produces similar changes in single neuron tuning and firing rate in the macaque prefrontal cortex during spatial WM tasks as systemically administered ketamine [10]. In addition, ketamine shows the greatest effects on prefrontal activity in imaging studies [8, 27]. One possibility is that changes in the architecture of LPFC circuits, such as expansion of layers 2/3 and increase in the size and number of spines on pyramidal cells with an abundance of NMDARs, makes the LPFC more vulnerable to the effects of ketamine relative to other areas. Indeed, the density of dendritic spines in pyramidal cells is higher in LPFC relative to LIP [38]. Although systemic administration of a drug may produce similar concentrations across brain vascular networks, idiosyncrasies in receptor distribution and their molecular regulation may allow heterogeneity of dose dependent local effects [39].

The effects of ketamine reported here resemble results of previous studies using NMDAR blockers that have examined changes in neuronal activity during cognitive tasks. For example, using MK-801, a specific NMDAR blocker, Wang et al. [10] reported reduced neuronal tuning during a spatial WM task. Homayoun and Moghaddam [30] also demonstrated differential effects of NMDAR blockage using MK-801 on narrow and BS cells in rodents, which are similar to what we report here using ketamine. Finally, Zick et al. [40] showed that phencyclidine reduced cognitive performance in macaque monkeys when administered systemically. The latter was accompanied by reduction in synchronous firing between neurons and reduced effective connectivity within prefrontal microcircuits.

We show that ketamine impaired the animals’ performance in the WM task. However, it did not do so in the perceptual task when animals had continuous visual access to the target. Moreover, ketamine neither impaired the ability of the animals to make saccades or navigate the virtual environment. These results suggest that in low doses, similar to the ones used in medical practice to treat depression [41], ketamine mainly affects mental representations. The latter corresponds with the common use of ketamine to mimic symptoms of schizophrenia [6, 7, 9, 10, 21]. Interestingly, WM deficits are one of the most prevalent symptoms of schizophrenia and are also hypothesized to result from NMDAR hypofunction, which may explain how ketamine so closely replicates symptoms of the disorder [6, 9, 42].

In our study, spatial tuning of pyramidal cells was diminished by an increase in responses to the least-preferred locations, so one could speculate that mental representations were not abolished by ketamine, but they became less precise or distorted. Indeed, our decoding analysis indicated less reliable neuronal population codes for discriminating between remembered locations (see confusion matrices in Fig. 3a). This may explain the documented cases of ketamine causing perceptual distortions and hallucinations, especially in cases with decreased feedforward input from sensory cortices and enhanced top-down feedback signaling emanating from prefrontal mental representations [5, 43]. Higher reliance on distorted representations may cause perceptual aberrations, explaining early descriptions of ketamine’s dissociative properties [4].

Ketamine continues to gain popularity for the treatment of conditions like depression [1, 3]. Patients with depression also suffer from WM deficits [44]. So how could ketamine improve WM in patients with depression but cause WM deficits in healthy subjects? One explanation is that the mechanism of WM deficits during depression are associated with a decrease in the overall activity of LPFC microcircuits mediated by a decrease in excitatory neurotransmission or an imbalance of inhibition/excitation [45]. We show that ketamine increases the level of activity of certain neuron types (e.g., BS excitatory cells). This increased activity may cause deficits in healthy subjects but ‘restore’ activity levels in patients with depression; however, this explanation requires specific testing. Nonetheless, our findings call for a careful evaluation on the impact of therapeutically administered ketamine on prefrontal cortex mediated cognition.

Finally, our results suggest that population codes for mental representations in LPFC rely on a delicate balance between the activation of excitatory and inhibitory neuron types mediated by NMDARs. A break-down of this balance may explain cognitive symptoms found in schizophrenia and other brain diseases exhibiting LPFC abnormalities and NMDAR hypoactivity [6, 9, 10, 42], as well as the disparate actions of ketamine on cognition and behavior.

## Materials and methods

Two adult male rhesus macaques (*Macaca mulatta*) were used in this experiment (age: 10, 9; weight: 12, 10 kg). We chose to use two animals in order to minimize the number of non-human primates used in the experiment and to ensure reproducibility between at least two animals. The *n* value for each analysis was determined individually as the smallest unit of observation, which was most often session. Results shown in the main text and figures represent results across subjects unless otherwise specified.

### Ethics statement

Animal care and handling including basic care, animal training, surgical procedures, and experimental injections were pre-approved by the University of Western Ontario Animal Care Committee. This approval ensures that federal (Canadian Council on Animal Care), provincial (Ontario Animals in Research Act), regulatory bodies (e.g., CIHR/NSERC), and other national standards (CALAM) for the ethical use of animals are followed. Regular assessments for physical and psychological well-being of the animals were conducted by researchers, registered veterinary technicians, and veterinarians.

### Task

The current task takes place in a virtual environment. This environment was developed using Unreal Engine 3 development kit, utilizing Kismet sequencing and UnrealScript (UDK, May 2012 release; Epic Games). More about this platform and the recording setup can be found in Doucet et al. [46] Within this virtual environment, target locations were arranged in a 3 × 3 grid and spaced 290 unreal units apart (time between adjacent targets is ~0.5 s). Movement speed was fixed throughout navigation.

### Experimental setup

The task was presented on a computer LDC monitor positioned 80 cm from the subjects’ eyes (27” ASUS, VG278H monitor, 1024 × 768 pixel resolution, 75 Hz refresh rate, screen height equals 33.5 cm, screen width equals 45 cm). Subjects performed the experiment in an isolated room with no illumination other than the monitor. The walls, doors, and ceiling of the room were RF shielded and contained no AC power lines. Cables providing power to the setup equipment entered the room through a small aperture in a wall and were shielded to minimize interference with the recordings. Eye positions were monitored using a video-oculography system with sampling at 500 Hz (EyeLink 1000, SR Research). A custom computer program-controlled the stimulus presentation (through Unreal Engine 3), reward dispensation, and recorded eye position signals and behavioral responses. Subjects performed the experiment while seated in a standard enclosed primate chair (Neuronitek) and were delivered juice reward through a tube attached to the chair and an electronic reward integration system (Crist Instruments). Prior to the experiments, subjects were implanted with custom fit, PEEK cranial implants which housed the head posts and recording equipment (Neuronitek). See Blonde et al. [47] for more information. The head posts were attached to a head holder to fix the monkeys’ heads to the primate chair during training and experimental sessions.

### Microelectrode array implant

Surgical procedures were conducted under general anesthesia induced by ketamine and maintained using isoflurane and propofol. Two 10 × 10, microelectrode Utah arrays (96 channels, 1.5 mm in length and separated by at least 0.4 mm) (Blackrock Microsystems) were chronically implanted in each animal. They were located in the left LPFC (anterior to the arcuate sulcus and on either side of the posterior end of the principal sulcus) [22]. Brain navigation for surgical planning was conducted using Brainsight (Rogue Research Inc.) (see Fig. S8a, b). Arrays were placed and impacted ~1.5 mm into the cortex. Reference wires were placed beneath the dura and a grounding wire was attached between screws in contact with the pedestal and the border of the craniotomy. Electrode placement was approximated using CT imaging post-operatively (Fig. S8c).

### Neuronal recordings and spike detection

Neuronal data was recorded using a Cerebus Neural Signal Processor (Blackrock Microsystems) via a Cereport adapter. The neural signal was digitized (16 bit) at a sample rate of 30 kHz. Spike waveforms were detected online by thresholding at 3.4 standard deviations of the signal. The extracted spikes were semi-automatically resorted with techniques utilizing Plexon Offline Sorter (Plexon Inc.). Sorting results were then manually supervised. Multiunits consisted of threshold-crossing events from multiple neurons with action potential-like morphology that were not isolated well enough to be classified as a well-defined single unit (for spike sorting example see Fig. S8d, e). We collected behavioral data across 18 ketamine-WM sessions (nine in animal T, nine in animal B) and neuronal data from 17 ketamine-WM sessions with one session from animal T removed due to incomplete synchronization of neuronal data during the recording. This yielded a total of 2906 units recorded during ketamine-WM sessions: 1814 single neurons (259 in animal T, 1555 in animal B) and 1092 multiunits (533 in animal T, 559 in animal B). Behavior and neuronal data was recorded from seven saline-WM sessions resulting in 1117 units in total: 674 single units (48 in animal T, 626 in animal B) 443 multiunits (126 in animal T, 317 in animal B). Behavioral data from four ketamine-perception sessions were analyzed (two in animal T, two in animal B).

### Ketamine injection

Both animals experienced all experimental conditions. The sessions in which either ketamine or saline was administered were randomized. Animals were trained to voluntarily receive injections in the primate chair while in the experimental setup. An intramuscular injection of either ketamine (0.25, 0.4, or 0.8 mg/kg) or saline (0.25 mg/kg) was administered in the hamstring muscles by a registered veterinary technician. The ketamine doses were titrated so they did not induce visible behavioral changes in the animals such as nystagmus or somnolence. Ketamine injections were spaced at least 2 days apart to allow for washout of the drug [28].

### Behavioral analysis

Correct trials are trials in which subjects reach the correct target location within 10 s. The percent of correct trials was compared to chance (11%) for each session using binomial tests.

The percent of correct trials over time was calculated using 15 equally sized trial bins for each injection period. The resulting 45 data points per session were averaged over all ketamine-WM and saline-WM sessions for each animal and then combined across subjects. Statistical analysis was conducted by comparing the percent of correct trials binned over the three injection periods (pre, early post, and late post-injection periods) for ketamine-WM and saline-WM sessions. Response time was calculated for correct trials as the duration between navigation onset and end of trial for each experimental condition (target location) for each recording session.

### Trajectory analysis

Analyses of animals’ trajectories within the navigation period are conducted on trials in which the animals cross a predetermined line that divides the start enclave from the main body of the task arena. The task environment was divided into a 5 × 5 grid containing 25 regional cells of equal dimensions. The grid was sized so that cells tightly enclose target locations (see Fig. 1j). For each trial, we calculated whether the subject entered each cell during navigation resulting in either 1 (entered) or 0 (not entered) per cell. The number of trials in which each cell was entered was then divided by the total number of trials. This resulted in a percent value for each cell for each target location condition (25*9 conditions, *n* = 225 per session) that was then averaged over all ketamine-WM or saline-WM sessions. We then calculated increases in average percent values for each cell between injection periods (values above 0 included).

### Spatial selectivity

Single units (2488 from 17 ketamine-WM and seven saline-WM sessions) were tested for selectivity for target location during a given epoch for all trials by computing a one-way analysis of variance on epoch-averaged firing rates with target location as the independent variable. A unit was defined as selective if the test resulted in *p* < 0.05. A neuron’s preferred location was defined as the location that elicited the largest response during the epoch of interest. The least-preferred location was defined as the location that elicited the smallest response.

To ensure consistent sample size between injection periods, we subsampled trials without replacement to the minimal number of trials between the pre, early post and late post-injection periods. This was repeated 50 times and the median values from all iterations was calculated. The proportion of tuned single units for each task epoch (cue and delay) were compared between injection periods for ketamine-WM and saline-WM sessions using Chi-Square tests.

### Ranked target selectivity

Neurons were ranked from their preferred to least-preferred location based on average firing rate. This was repeated for each injection period. We then calculated the slope of a linear regression model fitted to the ranked responses averaged across neurons in a session for each of the nine target locations. A higher negative slope indicates higher firing rate for preferred locations (higher ranked) compared to less preferred locations (lower ranked) which gives a proxy of tuning. This was calculated for each injection period in each session (*n* = 17).

### Spike density functions

The activity of tuned single neurons were plotted as trial averaged spike density functions (SDFs) for each task condition (target location) which were generated by convolving the spike train with a Gaussian kernel (standard deviation = 150 ms). We normalized responses by the maximum firing rate for each neuron’s preferred target location.

### Plane fitting

In order to visualize neuronal responses to different target locations within the 2D space, we fit a second order polynomial surface to the mean normalized firing rate for the 9 target location conditions to the x- and y- coordinates of each target location. Firing rate was normalized by the maximum firing rate in the ketamine pre-injection period. This method was used for visualization (Fig. 2a, b), not for quantitative analysis.

$$f\left( {x,y} \right) = {\mathrm{p}}_{0,0} + {\mathrm{p}}_{1,0}x + {\mathrm{p}}_{0,1}y + {\mathrm{p}}_{2,0}x^2 + {\mathrm{p}}_{0,2}y^2 + {\mathrm{p}}_{1,1}xy$$

### Neuronal ensemble decoding

We used a linear SVM (Libsvm 3.14) [48] with fivefold cross-validation to extract task-related activity from *z*-score normalized population-level responses using both single units and multiunits on a single trial basis. The regularization parameter used was the optimal penalty parameter *C* (refer to Eq. 1 in Fan et al. [48]). The classifiers used firing rates calculated over epoch durations (cue, 3000 ms; delay, 2000 ms; response first 2000 ms) from ensembles of neurons simultaneously recorded within each session to predict target location for correct and incorrect trials within the virtual arena (left, center, right).

For each session, we calculated decoding performance for neuronal ensembles with a maximum of 16 neurons since decoding performance plateaued around this point (see Fig. S3a, b). We began building ensembles by selecting the neuron with highest individual performance for decoding target location. This neuron was then paired with all remaining neurons to find the pair of neurons that maximized decoding performance. We then used this pair and combined it iteratively with all remaining neurons to find the best trio. This procedure was repeated until 16 neurons were reached.

We pooled target locations across depth in order to have a sufficient number of trials for training and testing the classifiers. We chose to combine trials based on target direction in the environment (left, center, right) based on observations that neurons tended to show more similar responses to targets located in the same direction compared to targets located at the same depth within the environment. Observations were balanced between classes using subsampling (without replacement) which was repeated 20 times.

We maintained the same neurons in ensembles (for ensembles of 16 neurons) and used the same procedure to calculate chance performance obtained by randomizing class labels (all other data features remained unaltered). We repeated this shuffling procedure 10 times for each session. Subsampling was conducted 20 times in each iteration. Using this procedure, the shuffled decoding accuracy for one ketamine-WM session from animal T was higher than expected by chance; therefore, this session was removed from the analysis. Decoding accuracy between injection periods for ketamine-WM and saline-WM sessions was compared for each neuronal ensemble size. We ran the decoding procedure a second time restricting to correct trials only (in sessions with a sufficient number of samples for cross-validation). Finally, a third decoding analysis was conducted using all 9 target locations from neuronal data on a single trial basis using SVM with fourfold cross validation (in sessions with a sufficient number of samples: one session in animal T, two sessions in animal B).

### Empirical decoding

Classification was performed using LDA with regularization on 1000 random neuronal ensembles of two, three, and five units using single units and multiunits (with firing rates >0.5 Hz). Decoding accuracy was determined using fivefold cross-validation. Since this analysis depends on binary data, trials (based on target locations) were grouped as being presented on the right or left of the environment (chance = 50%). Trials in which centrally placed targets are presented are not used in this analysis.

Further analysis was conducted using the top performing ensembles for each injection period (decoding accuracy in the 75th percentile).

### Theoretical decoding accuracy

Theoretical decoding accuracy (DPt) was calculated using the same 1000 random neuronal ensembles of two, three, and five units as the empirical decoding analysis and using previously described statistical properties of the population response [29]. DPt was calculated as:

$$DPt = {\Phi}\left( {\frac{1}{2}\left| {{\Delta}f} \right|\sqrt {\mathop {\sum}\nolimits_{i = 1}^N {\frac{{cos^2\hat \theta _i}}{{\hat \sigma _i^2}}} } } \right)$$

where ${\Phi}$ represents cumulative Gaussian of trial firing rates, the first term, |Δ*f* | represents the PS that measures target condition specific modulation of the population response (population tuning) and the second term, $\sqrt {\scriptstyle\mathop{\sum}\limits_{i = 1}^N {\frac{{cos^2\hat \theta _i}}{{\hat \sigma _i^2}}} }$, represents PP, which is a function of $\hat \theta _i$, the angle between the i-th eigenvector of the covariance matrix Σ and the direction of the stimulus $u_{{\Delta}f} = \frac{{{\Delta}f}}{{\left| {{\Delta}f} \right|}}$ vector tuning, and $\hat \sigma _i^2$ the i-th eigenvalue of the covariance matrix (see supplementary material for illustration). PP measures the population response variability (trial to trial variability). Further analysis was conducted using top performing ensembles (as classified by empirical decoding using LDA).

### Waveform classification

Single units were classified as either narrow (NS) or BS based on action potential width measured as peak-to-trough interval duration [31]. Average waveforms for each unit were interpolated with a cubic spline fit to increase the resolution of the data (×100). The duration between waveform peak and trough was then calculated based on time stamps from the minimal and maximal voltage values. Waveform widths for all neurons were plotted in a histogram. After removing outlier widths (>675 microseconds), 2314 units remained and are included in the analysis. A bimodal distribution was visualized and then quantified by fitting the data with either a single (1-Gaussian) or sum of two Gaussian functions (2-Gaussian) to determine optimal fit. The goodness of fit for both functions was determined using Akaike Information Criterion [49] with the lowest value determined for 2-Gaussians indicating bimodality.

The threshold dividing NS and BS (275 microseconds) was determined by setting a boundary at the inflection point of the two Gaussian fitted distributions (Fig. 4b) [31, 50]. Waveform amplitudes were normalized to the difference between the highest and lowest amplitudes for each unit waveform and waveforms were aligned at threshold crossing for visualization (Fig. 4a). Based on this threshold, 161 neurons were classified as NS and 2153 neurons were classified as BS. 750 delay tuned BS neurons were included for further analysis for ketamine-WM sessions and 246 delay tuned units were included for saline-WM sessions. 41 delay tuned narrow spiking neurons were included for ketamine-WM sessions and 11 delay tuned neurons were included for saline-WM sessions.

### Firing rate for preferred and non-preferred locations

Spike density functions (SDFs) using Gaussian kernels (150 ms std) were calculated for NS and BS neurons that were significantly tuned for target locations during the delay epoch (ANOVA, *p* < 0.1). We specifically obtained the SDFs for these neurons for their preferred and least-preferred locations during the delay epochs before ketamine or saline injection. We then calculated SDFs for these same locations in the post-injection period. Population activity was calculated by averaging SDFs between simultaneously recorded single units within the same electrode array and responses were normalized by the maximum population response. These population responses for each electrode array were then averaged over all ketamine-WM or saline-WM sessions. Firing rates were averaged during the delay epoch and were statistically compared using 1-tailed Wilcoxon Rank-sum tests between pre and early post-injection periods for preferred and least-preferred locations.

In addition, we used the same procedure as the preferred and least-preferred analysis but included responses to all target locations ranked from preferred (1) to least-preferred (9). We compared the firing rates from the pre-injection and post-injection periods for each target condition using 1-tailed Wilcoxon Rank-sum tests.

### Decoding eye position from neuronal data

We used a linear classifier (SVM) with fourfold cross validation to decode eye position on screen based on neuronal firing rates during fixations. Four target locations were selected as part of this analysis since their location on screen were easily separable. Four regions on the screen were outlined surrounding these target locations (Fig. S9b). Fixation periods occurring in either the cue or delay epoch that fell within these regions were used. Short fixation periods were removed (<6 ms). Firing rate was calculated for each neuron during each fixation period and was *z*-score normalized. Sessions missing observations (fixation periods) for 1 or more classes were excluded from this analysis. Sessions included: cue epoch, *n* = 11 sessions; delay epoch, *n* = 7 sessions. Neuronal populations included single units and multiunits.

### Gaze analysis

Gaze position was computed from eye tracking signals synchronized with the neuronal recordings and behavioral performance measurements [51]. The amount of time that gaze fell within the screen boundaries was calculated during the cue and delay epochs of the task and were statistically compared before and after ketamine or saline injection (Fig. S10a-f).

Eye movements were classified as saccades, fixations, or smooth pursuits based on previously published methods for eye movement classification in virtual environments in which periods of high acceleration approximate saccade epochs and movement patterns were used to determine precise saccade onset and offset. Foveations are classified as fixations or smooth pursuits based on measures of spatial range (see Corrigan et al. [51] for detailed method). The proportion of fixations falling within the trial specific target location compared to other potential target locations on the screen was calculated (Fig. S10g-l).

We calculated the total fixation time during the delay epoch as well as the fixation time on the trial specific target location for correct trials. We compared the proportion of fixation time on the target location related to all fixation time during delay (target location fixation duration / total fixation duration) between the three injection periods for ketamine and saline sessions using 2-way analysis of variance with injection period and drug (saline or ketamine) as factors.

### Decoding target location using eye position

During the cue and delay epochs, the screen was divided into 16 cells of equal dimensions. The number of foveations classified as fixations were calculated within each cell under the assumption that animals gather information from the virtual environment during such fixation periods [51]. We used a linear classifier (SVM) with fivefold cross-validation to determine whether target location could be predicted on a single trial basis by the number of fixations within each cell (i.e., the extent to which animals fixate in each part of the visual environment). This analysis was compared with a decoding analysis using neuronal ensembles utilizing the same number of features (16 neuron ensembles).

### Saccade selectivity

To calculate the proportion of single units tuned for eye position in both retinocentric and spatiocentric reference frames, we assessed saccade position in both retinocentric and screen centered coordinates. We used a quadrant binning pattern for a 40° × 30° field. To keep reference frames for a particular neuron consistent, we made sure that both reference frames had the same power by ordering the bins from highest saccade count to lowest, then pairing them across reference frames, and then dropping saccades from the bin that had more out of the pair. A bin had to have at least ten saccades to be acceptable and sessions had at least three acceptable bins. Neurons with sufficient data were then analyzed using Kruskal–Wallis analysis of variance.

### Statistics

Additional statistical information is outlined in Table. S1. See Fig. S11 for illustrated equation detailing the calculation of PS and PP.

## Supplementary information

Supplemental Figures

Supplemental Statistics Table

## Data Availability

Data supporting the findings of this study is available from the corresponding authors on reasonable request and will be fulfilled by MR.

## Code availability

MATLAB codes used the analyze the data are available from MR.
