# Supplementary material for: Ketamine disrupts naturalistic coding of working memory in primate lateral prefrontal cortex networks
Source: Mol Psychiatry. 2021 May 12;26(11):6688–703. doi: 10.1038/s41380-021-01082-5 (PMC8760073; doi:10.1038/s41380-021-01082-5)
Supplement: Supplementary file 1 — Supplemental Figures [file 41380_2021_1082_MOESM1_ESM.pdf]

Supplementary Material

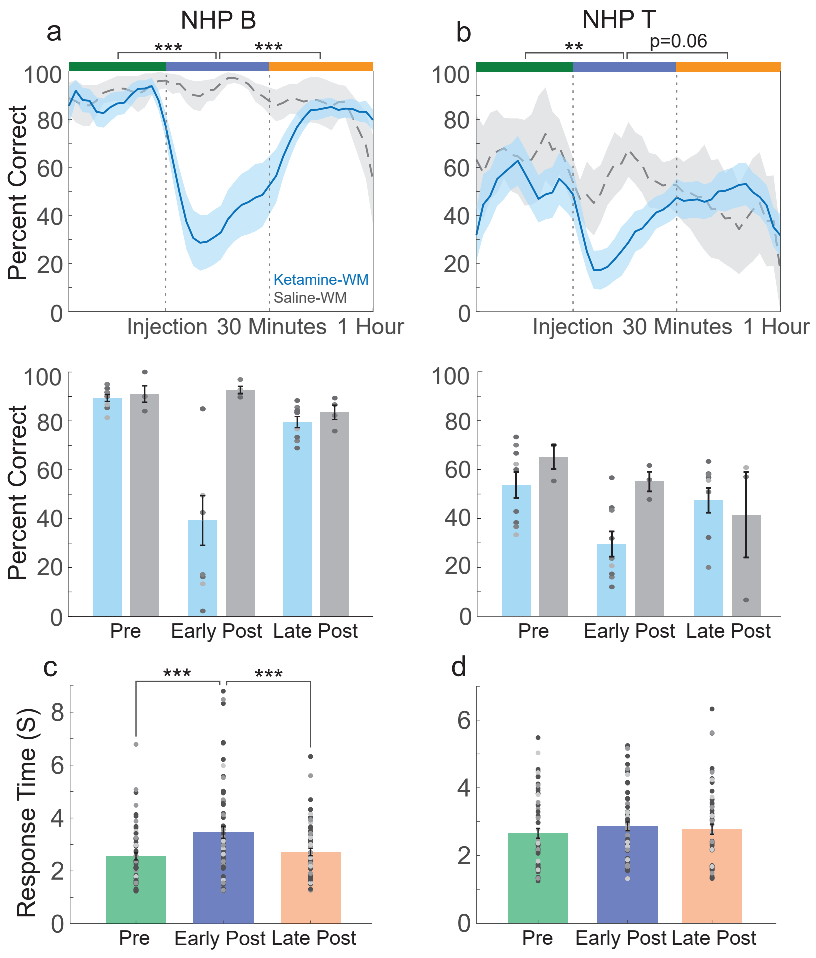

Supplementary Figure 1. Task performance per subject

a, Average percent of correct trials over ketamine-WM (blue) and saline-WM (grey) sessions for NHP B. **b**, Average percent of correct trials for ketamine-WM (blue) and saline-WM (grey) sessions for NHP T. **c**, Average response time for correct trials for NHP B for each injection period. **d**, Average response time for correct trials for NHP T for each injection period. All error bars are SEM. *<0.05, **<0.01, ***<0.001.

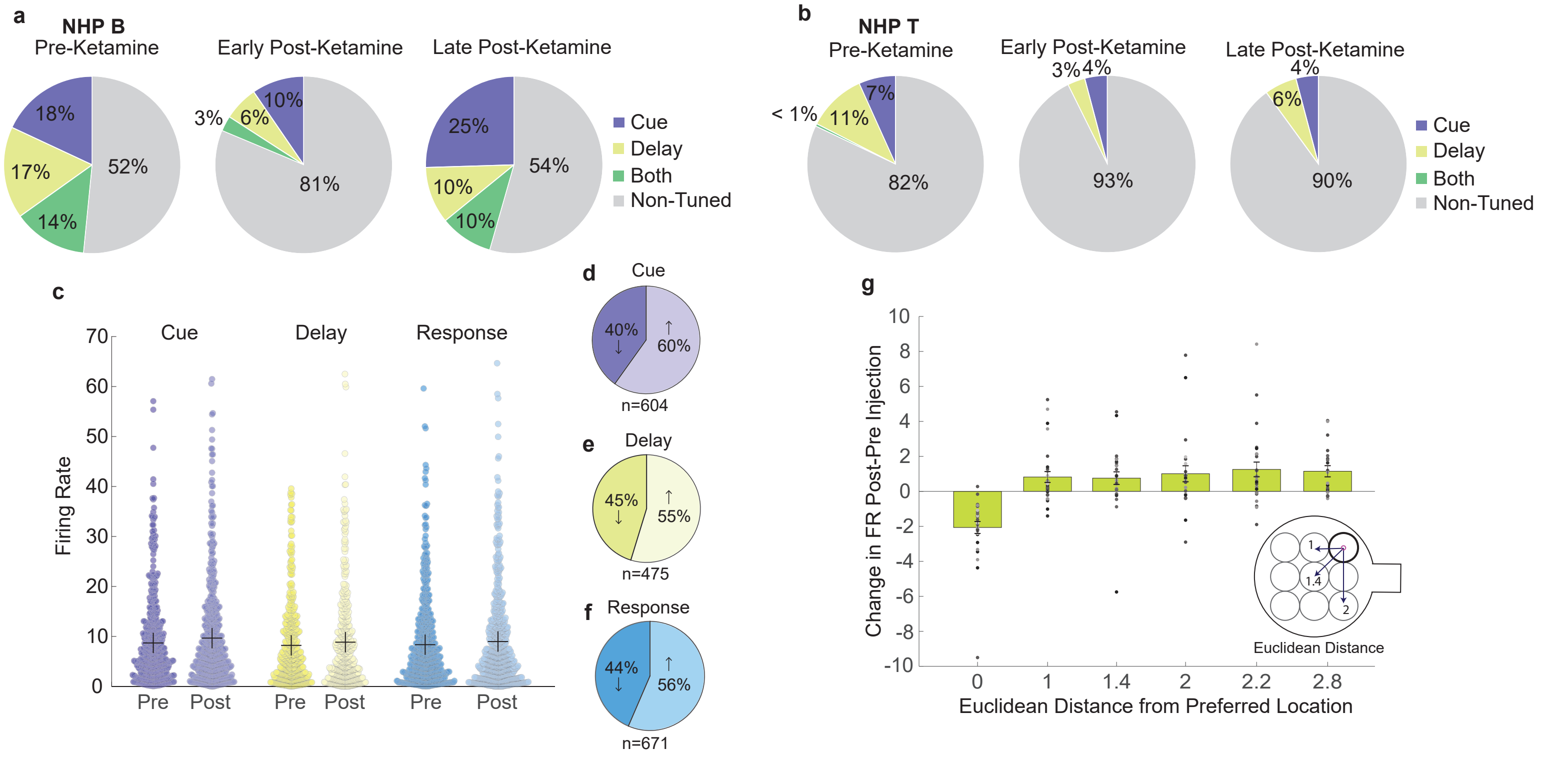

Supplementary Figure 2. Changes in neuron tuning and firing rate

a, Average proportion of tuned single units for ketamine-WM sessions for NHP B during the cue, delay, or both epochs. **b**, Average proportion of tuned single units for ketamine-WM sessions for NHP T for the trial epochs. **c**, Difference in firing rate of single units for correct trials during cue, delay, and response epochs between pre and early post-injection periods for ketamine-WM sessions. Mean indicated by '+'. **d**, Proportion of cue tuned single units showing either an increase or decrease in firing rate after ketamine injection during the cue epoch for correct trials. **e**, Proportion of delay tuned single units showing either an increase or decrease in firing rate after ketamine injection during the delay epoch for correct trials. **f**, Proportion of response tuned single units showing either an increase or decrease in firing rate after ketamine injection during the first 2000 ms of the response epoch for correct trials. **g**, Average change in firing rate for single units from the pre-injection to the early post-injection period as a function of Euclidean distance from neuron's original preferred location during the delay epoch for each target location for ketamine-WM sessions. Euclidean distance of 0 indicates preferred location and data points represent the average of each array for each session. Error bars are SEM.

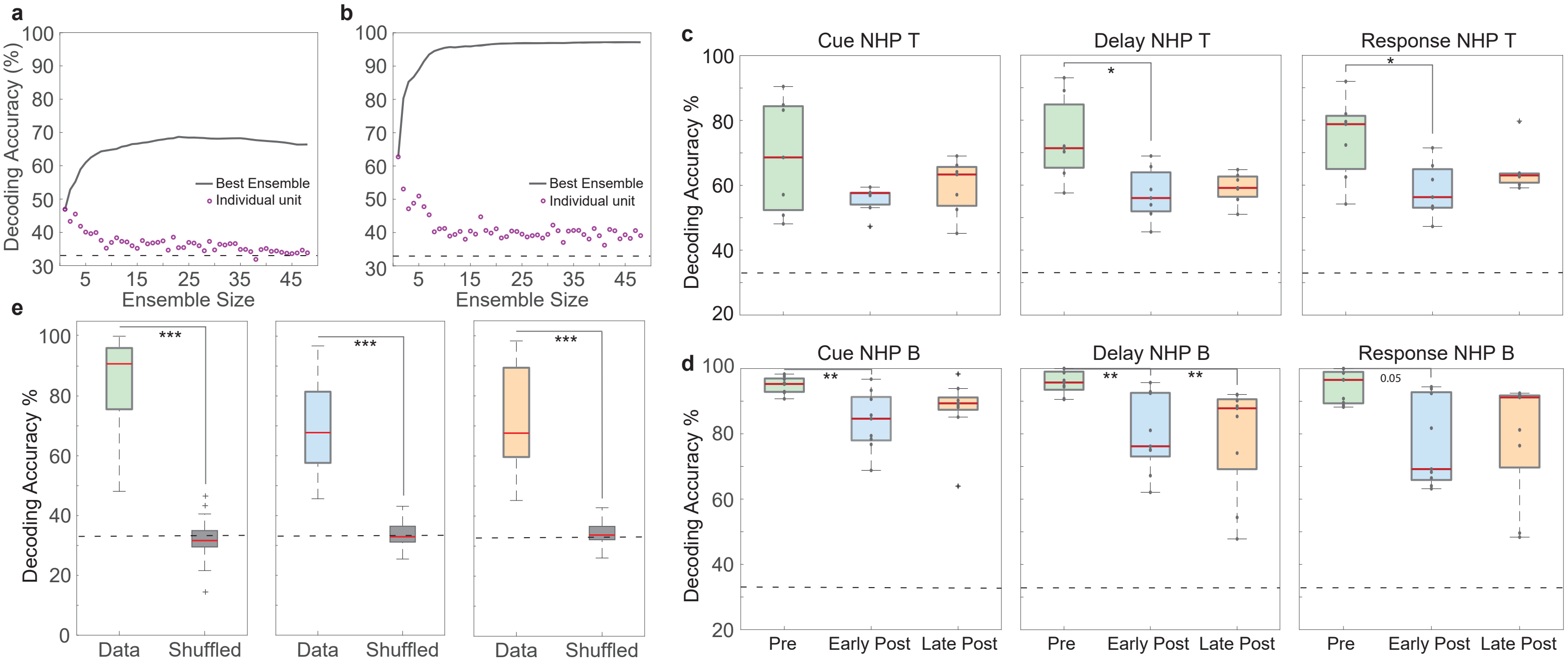

Supplementary Figure 3. Decoding performance per subject

a, Single NHP T session example of decoding accuracy as a function of neuronal ensemble size. **b**, Single NHP B session example of decoding accuracy as a function of neuronal ensemble size. Purple data points represent individual neuron contribution to decoding accuracy. **c**, Sixteen neuron ensemble decoding accuracy for trial epochs over injection periods for ketamine-WM sessions for NHP T. **d**, Sixteen neuron ensemble decoding accuracy for trial epochs over injection periods for ketamine-WM sessions for NHP B. Data points represent decoding accuracy per session. **e**, Decoding accuracy for ketamine-WM sessions with data combined between trial epochs for injection periods. Decoding accuracy for shuffled data in grey (shuffled trial condition labels). Red center lines indicate median, the bottom and top edges of the box indicate the 25th and 75th percentiles. The whiskers extend to non-outlier data points (approximately within 2.7 std) and the outliers are plotted using '+'. *<0.05, **<0.01, ***<0.001.

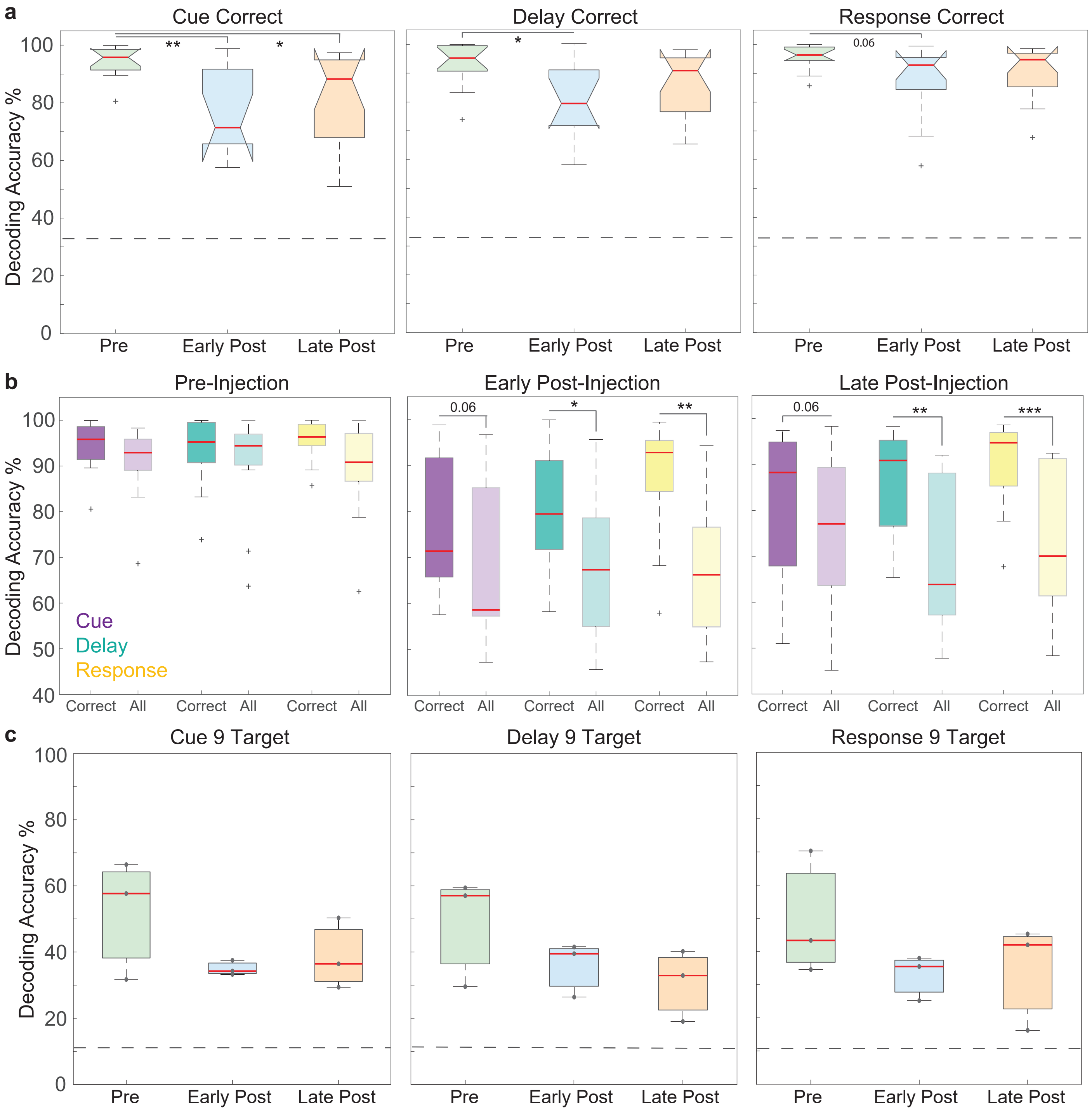

Supplementary Figure 4. Ensemble decoding for correct trials and 9 target locations

a, Decoding target location from neuronal ensembles using correct trials. Decoding accuracy for ketamine-WM sessions for pre-injection (green), early post-injection (blue), and late post-injection periods (orange) for trial epochs. Chance performance is indicated by dashed grey line. **b**, Comparison between decoding accuracy using correct trials and using all trials for trial epochs and injection periods. **c**, Decoding nine target locations from neuronal ensembles. Decoding accuracy for ketamine-WM sessions for pre-injection (green), early post-injection (blue), and late post-injection periods (orange) (n=3 sessions) for trial epochs. Data points represent decoding accuracy per session. Chance performance is indicated by dashed grey line. Red center lines indicate median, the bottom and top edges of the box indicate the 25th and 75th percentiles. The whiskers extend to non-outlier data points (approximately within 2.7 std) and the outliers are plotted using '+'. *<0.05, **<0.01, ***<0.001.

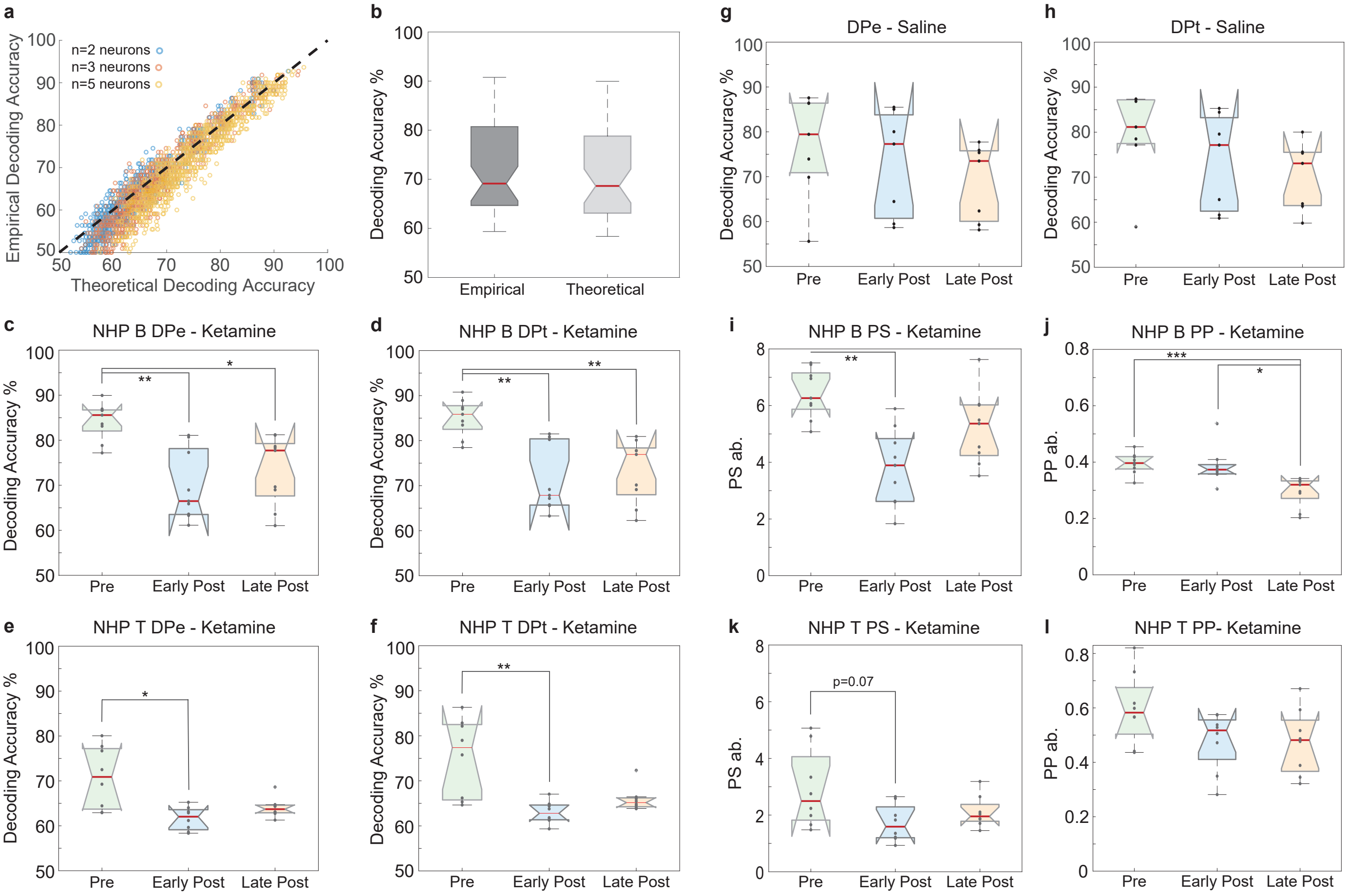

Supplementary Figure 5. Empirical and theoretical decoding per subject

a, Example session showing empirical and theoretical decoding accuracy per neuronal ensemble with decoding above chance level (50%). **b**, Comparison between empirical (DPe) and theoretical (DPt) decoding accuracy for ketamine-WM sessions combined between epochs for the pre-injection period. **c**, DPe for ketamine-WM sessions from NHP B. **d**, DPt for ketamine-WM sessions from NHP B. **e**, DPe for ketamine-WM sessions from NHP T. **f**, DPt for ketamine-WM sessions from NHP T. **g**, DPe for saline-WM sessions. **h**, DPt for saline-WM sessions. **i**, PS for ketamine-WM sessions for NHP B. **j**, PP for ketamine-WM sessions for NHP B. **k**, PS for ketamine-WM sessions for NHP T. **l**, PP for ketamine-WM sessions for NHP T. Data points represent values per session. Red center lines indicate median, the bottom and top edges of the box indicate the 25th and 75th percentiles. The whiskers extend to non-outlier data points (approximately within 2.7 std) and the outliers are plotted using '+'. *<0.05, **<0.01, ***<0.001.

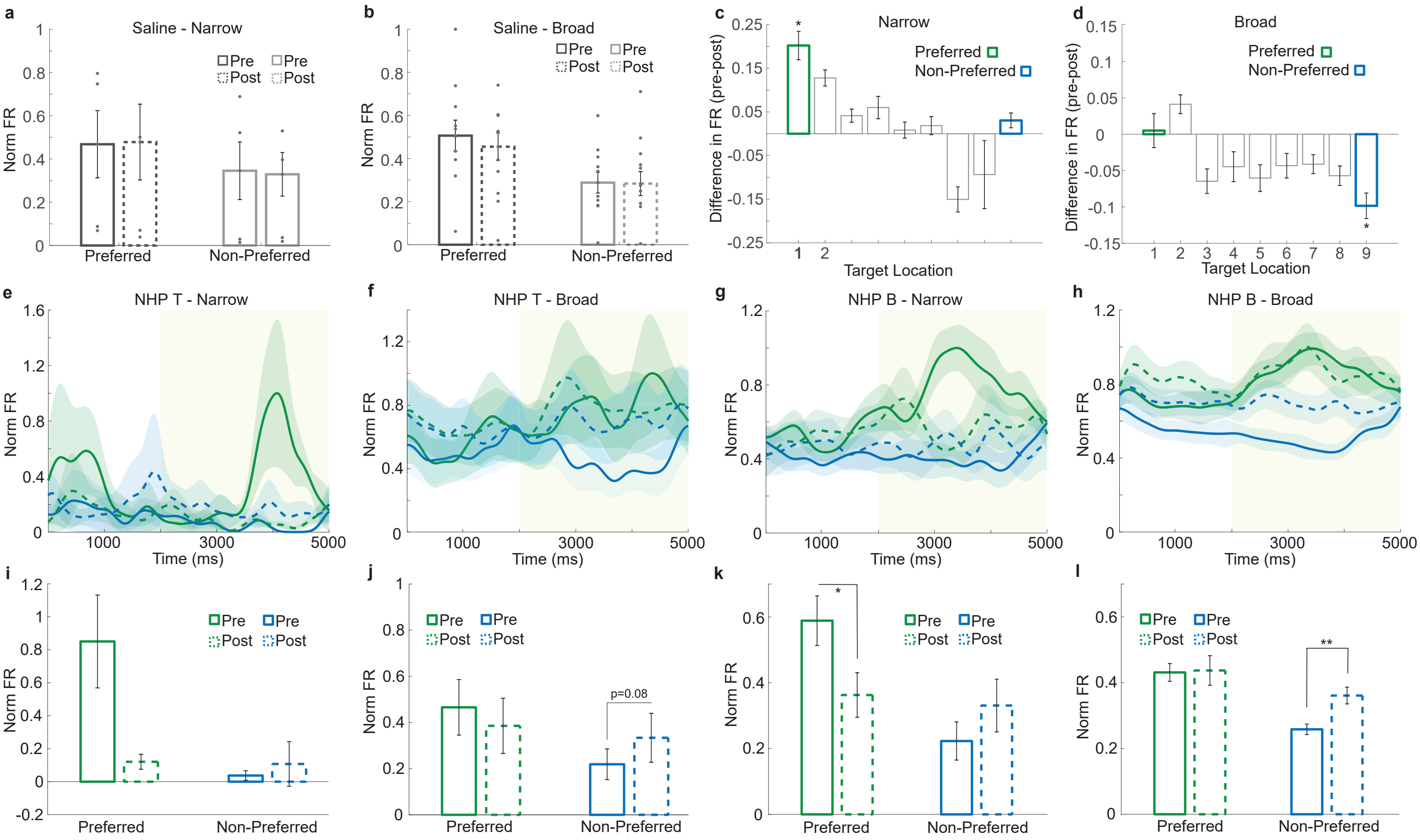

Supplementary Figure 6. Changes in narrow and broad neuron firing rates per subject

a, Firing rates for saline-WM sessions for narrow spiking neurons averaged over the delay epoch for preferred and least-preferred locations. **b**, Firing rates for saline-WM sessions for broad spiking neurons averaged over the delay epoch for preferred locations and least-preferred locations. Data points represent values per electrode array for each session. **c**, Difference in firing rate (pre-post in-jection) for delay tuned narrow spiking neurons for target locations ranked from preferred to least-preferred. **d**, Difference in firing rate (pre-post injection) for delay tuned broad spiking neurons for target locations ranked from preferred to least-preferred. **e**, Population SDFs for cue and delay (yellow) epochs for delay tuned narrow spiking neurons for NHP T ketamine-WM sessions. **f**, Population SDFs for cue and delay (yellow) epochs for delay tuned broad spiking neurons for NHP T ketamine-WM sessions. **g**, Population SDFs for cue and delay (yellow) epochs for delay tuned narrow spiking neurons for NHP B ketamine-WM sessions. **h**, Population SDFs for cue and delay (yellow) epochs for delay tuned broad spiking neurons for NHP B ketamine-WM sessions. **i**, Firing rates for narrow spiking neurons averaged over the delay epoch for preferred and least-preferred locations for pre and early post-injection periods for NHP T ketamine-WM sessions. **j**, Firing rates for broad spiking neurons averaged over the delay epoch for preferred and least-preferred locations for pre and early post-injection periods for NHP T ketamine-WM sessions. **k**, Firing rates for narrow spiking neurons averaged over the delay epoch for preferred and least-preferred locations for pre and early post-injection periods for NHP B ketamine-WM sessions. **l**, Firing rates for broad spiking neurons averaged over the delay epoch for preferred and least-preferred locations for pre and early post-injection periods for NHP B ketamine-WM sessions. Error bars are SEM. *<0.05, **<0.01, ***<0.001.

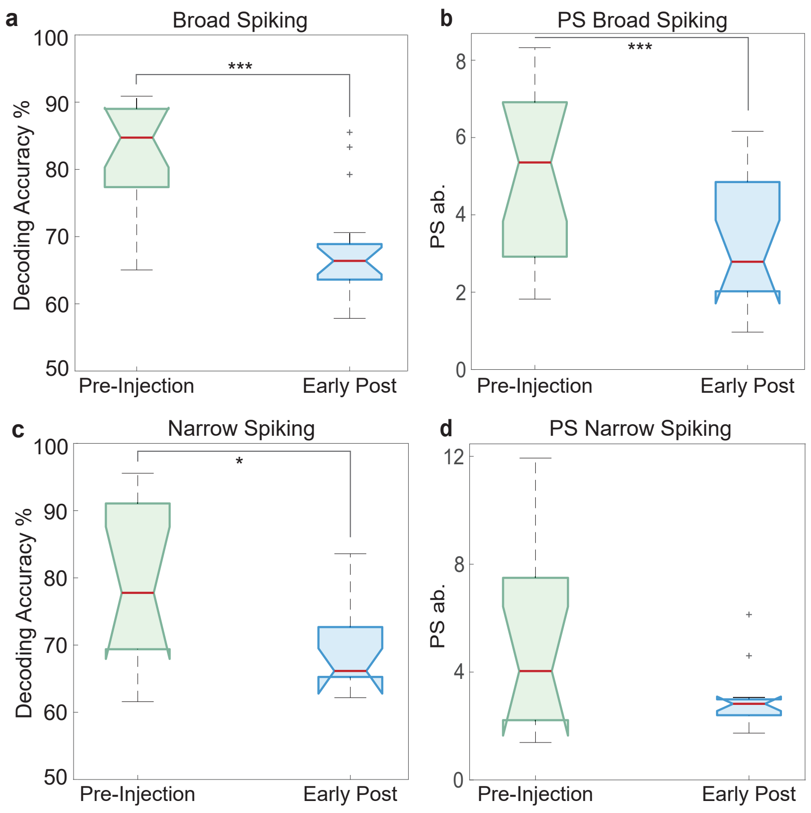

Supplementary Figure 7. Theoretical decoding and population signal for narrow and broad spiking neurons

a, DPt of target location (left/right) from neuronal ensembles of broad spiking neurons. Decoding accuracy over the delay epoch for ketamine-WM sessions (n=16) compared between pre and early post-injection periods. **b**, PS for ketamine-WM sessions for broad spiking neuronal ensembles compared between pre and early post-injection periods. **c**, DPt of target location from neuronal ensembles of narrow spiking neurons. Decoding accuracy over the delay epoch for ketamine-WM sessions (n=12) compared between pre and early post-injection periods. **d**, PS for ketamine-WM sessions using narrow spiking neurons compared between pre and early post-injection periods. Red center lines indicate median, the bottom and top edges of the box indicate the 25th and 75th percentiles. The whiskers extend to non-outlier data points (approximately within 2.7 std) and the outliers are plotted using '+'. *<0.05, **<0.01, ***<0.001.

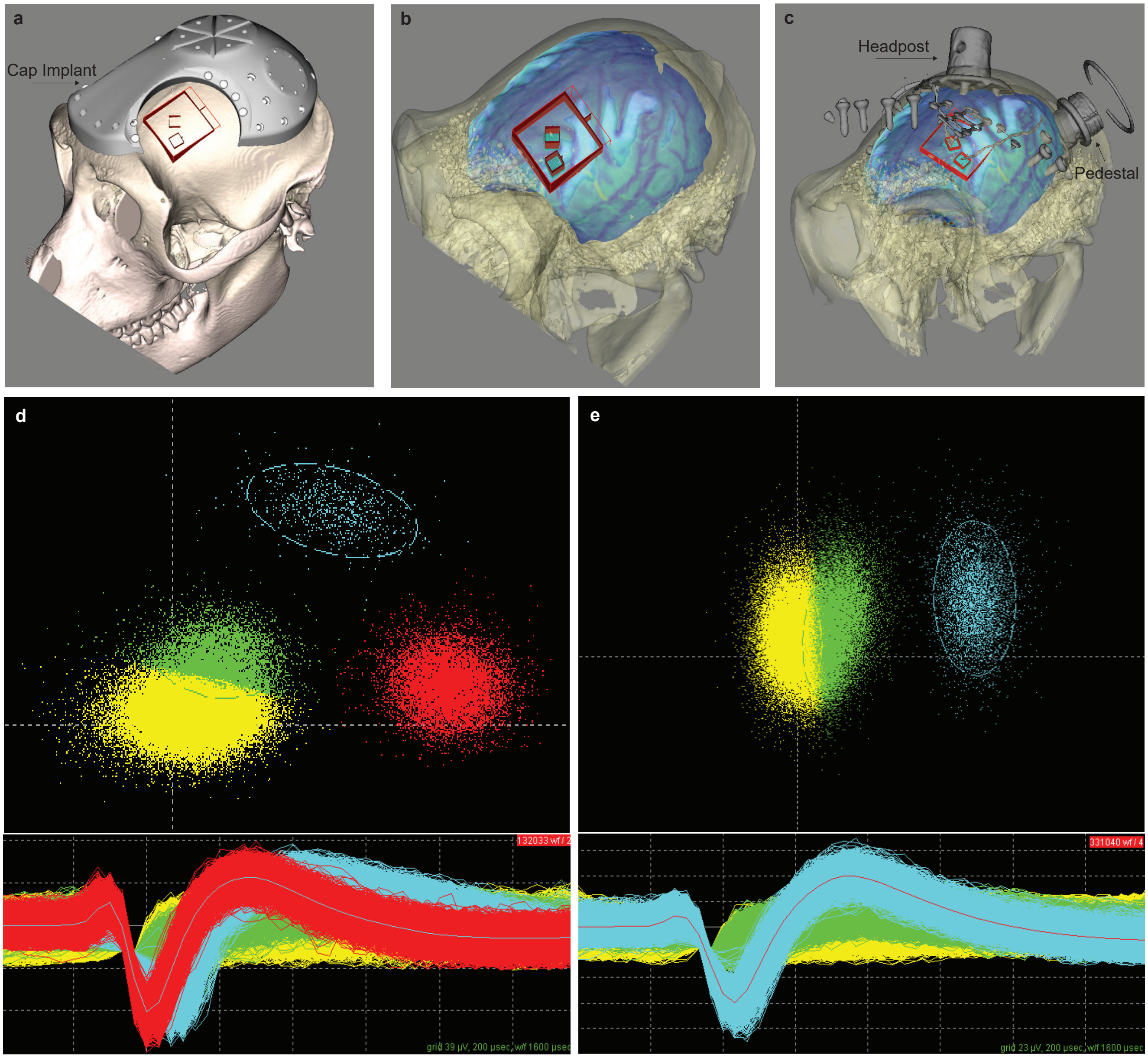

Supplementary Figure 8. Neural recording and spike sorting

a, 3D modeled skull using CT scan from NHP B showing pre-surgical planning conducted in BrainSight. Cap implant depicted as well as planned craniotomy site and planned array implantation site in reference to cap implant. **b**, 3D modeled skull and brain using CT scan and MRI from NHP B showing pre-surgical planning. **c**, 3D modeled skull and brain with overlaid CT imaged cap implant attachments, bone screws, and Utah arrays positioned approximately over the planned array implantation sites. **d**, Spike sorting example from NHP B using Plexon offline sorter. For our analysis, yellow waveforms are considered noise, the green unit would be classified as a multiunit and the blue and red units would be classified as single units. **e**, Spike sorted neuron example from NHP T. The green unit would be classified as a multiunit and the blue unit would be classified as a single unit.

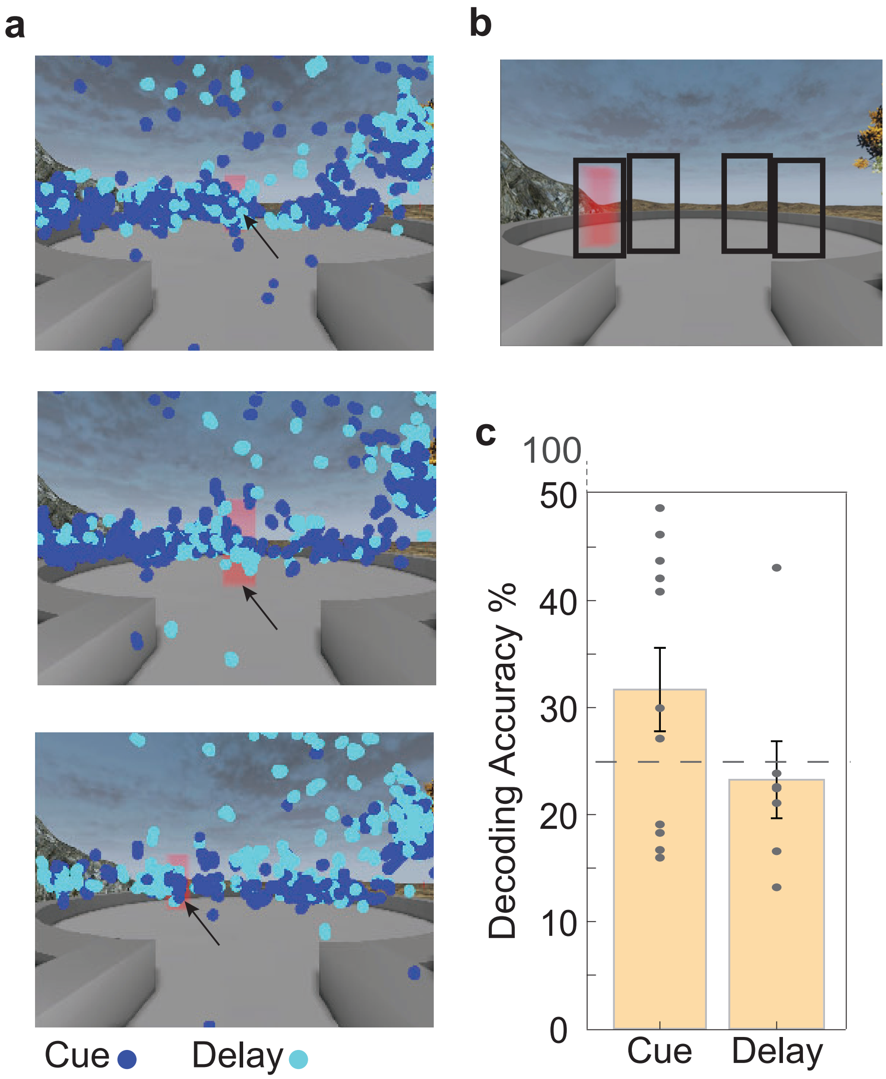

Supplementary Figure 9. Eye behavior

a, Fixation eye positions on screen during the cue and delay period of an example session. **b**, Four target locations outlined on the screen. **c**, Decoding accuracy for decoding eye position between four target locations on screen illustrated in panel b from neural activity during periods of fixation in the cue and delay period. Error bars are SEM.

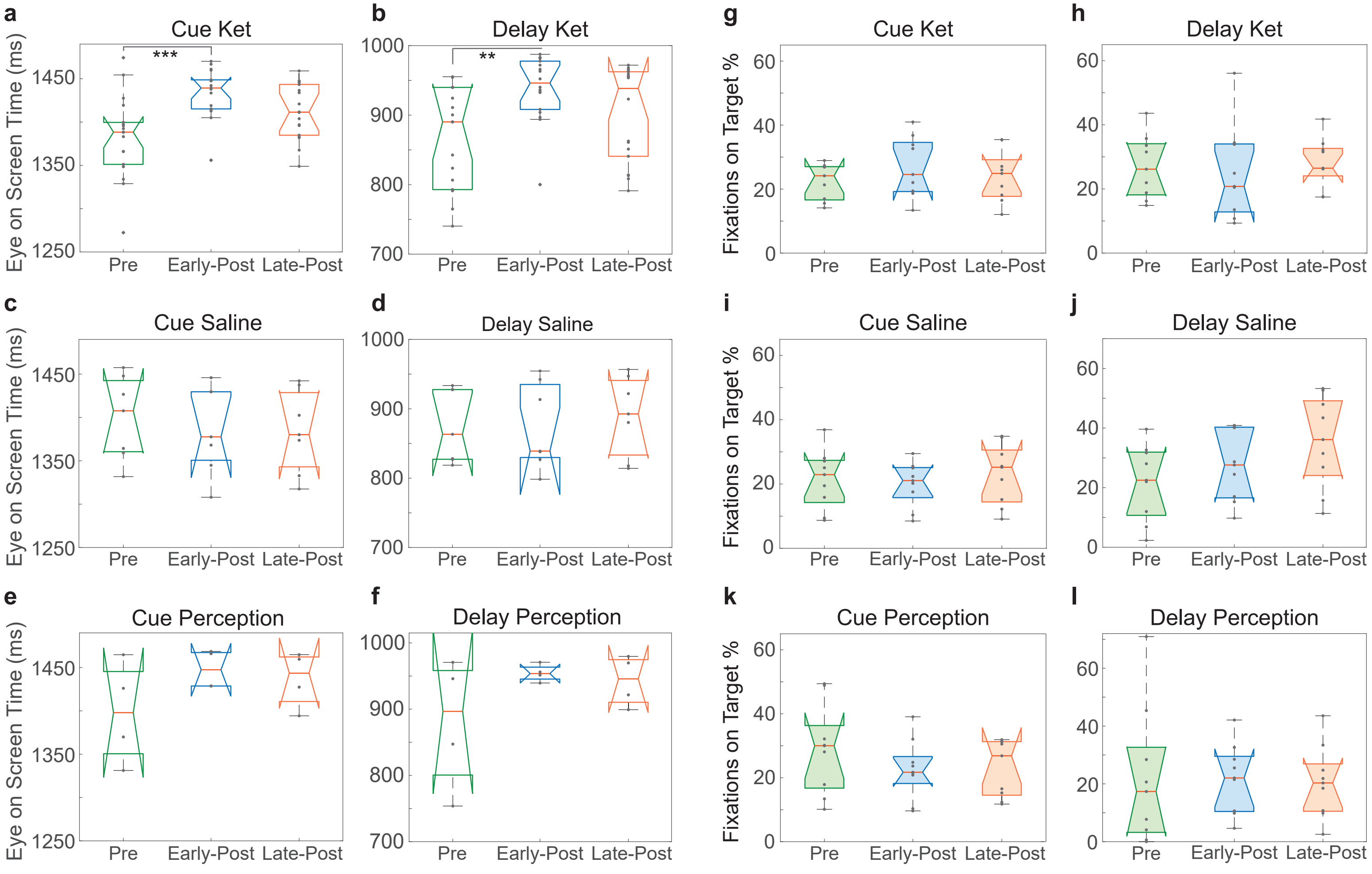

Supplementary Figure 10. Gaze behavior

a, Duration of eyes on screen during the cue epoch for ketamine-WM sessions. **b**, Duration of eyes on screen during the delay epoch for ketamine-WM sessions. **c**, Duration of eyes on screen during the cue epoch for saline-WM sessions. **d**, Duration of eyes on screen during the delay epoch for saline-WM sessions. **e**, Duration of eyes on screen during the cue epoch for ketamine-perception sessions. **f**, Duration of eyes on screen during the delay epoch for ketamine-perception sessions. Data points represent values per session. **g**, Percentage of fixations on target location during the cue epoch for ketamine-WM sessions. **h**, Percentage of fixations on target location during the delay epoch for ketamine-WM sessions. **i**, Percentage of fixations on target location during the cue epoch for saline-WM sessions. **j**, Percentage of fixations on target location during the delay epoch for saline-WM sessions. **k**, Percentage of fixations on target location during the cue epoch for ketamine-perception sessions. **l**, Percentage of fixations on target location during the delay epoch for ketamine-perception sessions. Data points represent values per target location condition. Red center lines indicate median, the bottom and top edges of the box indicate the 25th and 75th percentiles. The whiskers extend to non-outlier data points (approximately within 2.7 std) and the outliers are plotted using '+'. *<0.05, **<0.01, ***<0.001.

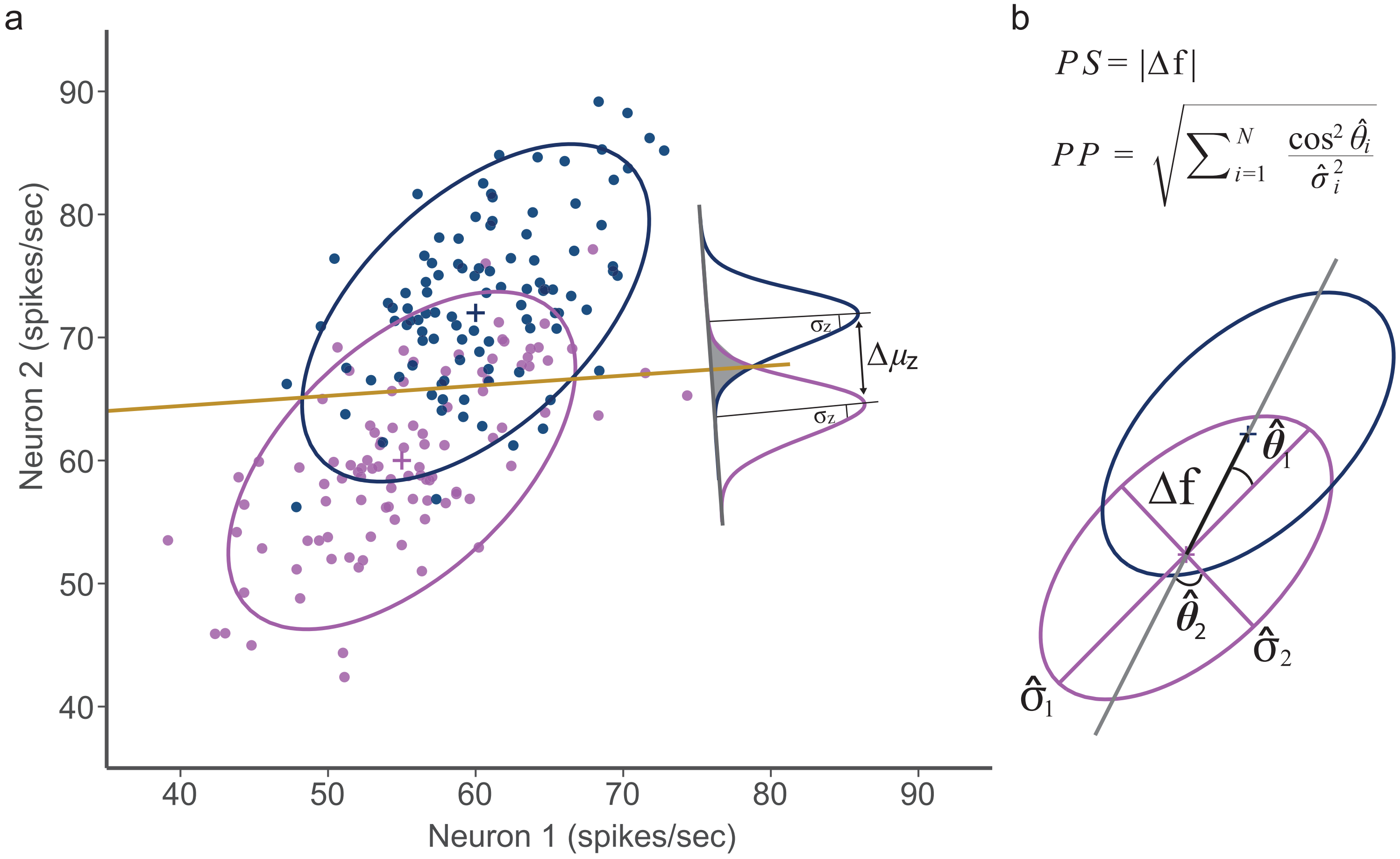

Supplementary Figure 11. Illustration of population signal and projected precision

a, Simulated data illustrates firing rates for two neurons. The covariance matrix and mean activity of the population of neurons determine the shape and location of the ellipsoid for target location 1 (purple) and target location 2 (blue) whereas the yellow line represents a linear classifier that divides the two clouds of data points (thus classifying target location). The difference between mean firing rate for the two target locations is represented by $\Delta\mu_z$ and standard deviation by σ_z **b**, Illustration of population signal and projected precision. PS is shown as the distance between the mean neural response for target location 1 and target location 2 (distance between the centers of the two ellipsoids as shown by the black line connecting '+' symbols). PP is then calculated from the angles $\hat{\theta}_i$ (i.e. $\hat{\theta}_1$ and $\hat{\theta}_2$) between each eigenvector of the covariance matrix and the stimulus tuning vector Δf as well as from their eigenvalues (length of each axis, $\hat{\sigma}_i^2$).

Supplementary Videos

Online Movie S1. Working Memory Task. https://www.youtube.com/watch?v=nZDYJw2aFLQ

One example trial of the working memory task. Featured: Cue, delay, and response epochs. Monkey's eye position in the virtual environment are indicated by the moving white circle with label of objects that falls within foveated position.

Online Movie S2. Ketamine's Effect on Task Performance. https://youtu.be/r5ouvtSx_XQ

Example trials of the working memory task before and after ketamine injection. Featured: Cue, delay, and response epochs. Monkey's eye position in the virtual environment are indicated by the moving white circle with label of objects that falls within foveated position.
