# Supplementary material for: Ketamine disrupts naturalistic coding of working memory in primate lateral prefrontal cortex networks
Source: Mol Psychiatry. 2021 May 12;26(11):6688–703. doi: 10.1038/s41380-021-01082-5 (PMC8760073; doi:10.1038/s41380-021-01082-5)
Supplement: Supplementary file 2 — Supplemental Statistics Table [file 41380_2021_1082_MOESM2_ESM.pdf]

Supplementary Table 1. Statistical analysis

All post hoc testing used the Tukey-Kramer method, controlling for multiple comparisons. All statistical tests are two-sided unless otherwise specified.

Figure	Sample Size (numbers indicate sessions unless otherwise stated)	Statistical Test	Values
Task performance (percent correct) compared to chance (11%)	Ketamine-WM<br>18<br><br>Saline-WM<br>7<br><br>Ketamine-Perception<br>4	Binomial test	**Pre-Injection:**<br>Ketamine-WM sessions<br>p<0.001<br>Saline-WM sessions<br>p<0.001<br>Ketamine-Perception sessions<br>p<0.001<br><br>**Early Post-Injection:**<br>Ketamine-WM sessions<br>13 sessions, p<0.05<br>Saline-WM sessions<br>p<0.0001<br>Ketamine-Perception sessions<br>p<0.0001<br><br>**Late Post-Injection:**<br>Ketamine-WM sessions<br>p<0.05<br>Saline-WM sessions<br>6 sessions, p<0.05<br>Ketamine-Perception sessions<br>p<0.05
**Fig. 1g**<br>Task performance (percent correct) compared between	Ketamine-WM<br>18<br><br>Saline-WM<br>7		**Ketamine-WM:**<br>Pre-Injection, *mean*=72, *median*=77<br>Early Post-Injection, *mean*=34, *median*=28<br>Late Post-Injection, *mean*=64, *median*=66<br><br>**Saline-WM:**<br>Pre-Injection, *mean*=80, *median*=84<br>Early Post-Injection, *mean*=77, *median*=90

injection periods		Two-way analysis of variance with post hoc pairwise comparisons	Late Post-Injection, *mean*=66, *median*=76<br><br>Drug: F=9.57, p=0.003, df=1,69<br>Injection Period: F=4.3, p=0.017, df=2,69<br>Interaction: F=4.85, p=0.011, df=2,69<br><br>Ket pre-injection, Sal pre-injection, p=0.9568<br>Ket pre-injection, Ket early post-injection, p=0<br>Ket pre-injection, Sal early post-injection, p=0.9958<br>Ket pre-injection, Ket late post-injection, p=0.8788<br>Ket pre-injection, Sal late post-injection, p=0.9893<br>Sal pre-injection, Ket early post-injection, p=0.0002<br>Sal pre-injection, Sal early post-injection, p=0.9997<br>Sal pre-injection, Ket late post-injection, p=0.5528<br>Sal pre-injection, Sal late post-injection, p=0.8236<br>Ket early post-injection, Sal early post-injection, p=0.0008<br>Ket early post-injection, Ket late post-injection, p=0.0023<br>Ket early post-injection, Sal late post-injection, p=0.0261<br>Sal early post-injection, Ket late post-injection, p=0.7666<br>Sal early post-injection, Sal late post-injection, p=0.9352<br>Ket late post-injection, Sal late post-injection, p=0.9999
	Ketamine-Perception<br>4	Repeated measures analysis of variance	**Ketamine-Perception:**<br>Pre-Injection, *mean*=79, *median*=84<br>Early Post-Injection, *mean*=84, *median*=90<br>Late Post-Injection, *mean*=85, *median*=93<br><br>F=0.25, p=0.786, df=2,6
**Fig. 1h** Response time compared between injection periods	Ketamine-WM<br>18<br>*n*=126 (values calculated per target location for conditions with trials in all injection periods)	Repeated measures analysis of variance with post hoc pairwise comparisons	**Ketamine-WM:**<br>Pre-Injection, *mean*=2.6, *median*=2.4<br>Early Post-Injection, *mean*=3.2, *median*=2.9<br>Late Post-Injection, *mean*=2.7, *median*=2.5<br><br>F=16.81, p<0.0001, df=2,250<br>Post Hoc<br>Early Post-Injection, Pre-Injection<br>p<0.0001<br>Late Post-Injection, Pre-Injection<br>p=0.330<br>Early Post-Injection, Late Post-Injection<br>p<0.0001

	Saline-WM<br>7<br>*n*=55<br><br>Ketamine-Perception<br>4<br>*n*=31		**Saline-WM:**<br>Pre-Injection, *mean*=2.6, *median*=2.4<br>Early Post-Injection, *mean*=2.7, *median*=2.5<br>Late Post-Injection, *mean*=2.7, *median*=2.4<br><br>F=1.71, p=0.186, *df*=2,108<br><br>**Ketamine-Perception:**<br>Pre-Injection, *mean*=2.5, *median*=2.3<br>Early Post-Injection, *mean*=2.5, *median*=2.5<br>Late Post-Injection, *mean*=2.5, *median*=2.5<br><br>F=0.22, p=0.800, *df*=2,60
**Fig. 1l**<br>Navigation in environment (difference in cells entered)	NHP T<br>8<br>Number of cells (25) * number of target locations with trials<br>Early post injection – pre-injection<br>Ketamine, *n*=154<br>Saline, *n*=161<br>Late post-injection – pre-injection<br>Ketamine, *n*=124<br>Saline, *n*=125<br>Early post-injection – late post-injection<br>Ketamine, *n*=159	Two-way analysis of variance with interaction effect with post hoc comparisons	**NHP T:**<br>**Ketamine**<br>Early Post-Injection – Pre-Injection<br>*mean*=6.9, *median*=4.2<br>Late Post-Injection – Pre-Injection<br>*mean*=3.3, *median*=0.9<br>Early Post-Injection – Late Post-Injection<br>*mean*=6.6, *median*=2.9<br><br>**Saline**<br>Early Post-Injection – Pre-Injection<br>*mean*=3.2, *median*=0<br>Late Post-Injection – Pre-Injection<br>*mean*=4.8, *median*=0<br>Early Post-Injection – Late Post-Injection<br>*mean*=2.5, *median*=0<br><br>**Ketamine and Saline Comparison**<br>Epoch, F=0.97, p=0.380, *df*=2,832<br>Drug, F=12.1, p=0.0005, *df*=1,832<br>Interaction, F=8.73, p=0.0002, *df*=2,832<br><br>Post Hoc<br>Early Post-Injection, Pre-Injection<br>p=0.002<br>Late Post-Injection, Pre-Injection<br>p=0.717

	Saline, $n=115$<br><br>NHP B<br>9<br>Number of cells (25) * number of target locations with trials<br>Early post injection – pre-injection<br>Ketamine, $n=162$<br>Saline, $n=158$<br>Late post-injection – pre-injection<br>Ketamine, $n=163$<br>Saline, $n=157$<br>Early post-injection – late post-injection<br>Ketamine, $n=134$<br>Saline, $n=145$<br><br>Ketamine-Perception<br>4<br>Early post injection – pre-injection<br>Perception, $n=197$<br>Late post-injection –		Early Post-Injection, Late Post-Injection<br>$p=0.001$<br><br>**NHP B:**<br>**Ketamine**<br>Early Post-Injection – Pre-Injection<br>$mean=4.6$, $median=2.1$<br>Late Post-Injection – Pre-Injection<br>$mean=3.6$, $median=1.8$<br>Early Post-Injection – Late Post-Injection<br>$mean=4.1$, $median=2.3$<br><br>**Saline**<br>Early Post-Injection – Pre-Injection<br>$mean=2.6$, $median=1.2$<br>Late Post-Injection – Pre-Injection<br>$mean=3.4$, $median=1.4$,<br>Early Post-Injection – Late Post-Injection<br>$mean=2.4$, $median=0.8$<br><br>**Ketamine and Saline Comparison**<br>Epoch, $F=0.51$, $p=0.604$, $df=2,913$<br>Drug, $F=15.16$, $p=0.0001$, $df=1,913$<br>Interaction, $F=3.4$, $p=0.034$, $df=2,913$<br><br>Post Hoc<br>Early Post-Injection, Pre-Injection<br>$p=0.004$<br>Late Post-Injection, Pre-Injection<br>$p=1$<br>Early Post-Injection, Late Post-Injection<br>$p=0.044$<br><br>**Perception**<br>Early Post-Injection – Pre-Injection<br>$mean=1.8$, $median=0$<br>Late Post-Injection – Pre-Injection<br>$mean=2.2$, $median=0$<br>Early Post-Injection – Late Post-Injection

	pre-injection Perception, n=194<br>Early post-injection – late post-injection Perception, n=203<br><br>Ketamine-WM<br>17		*mean*=2.0, *median*=0<br><br>**Ketamine-WM and Ketamine-Perception Comparison**<br><br>Epoch, F=3.09, p=0.046, df=2,1022<br>Drug, F=25.6, p=0, df=1,1022<br>Interaction, F=5.26, p=0.005, df=2,1022<br><br>Post Hoc<br>Early Post-Injection, Pre-Injection<br>p=0<br>Late Post-Injection, Pre-Injection<br>p=0.999<br>Early Post-Injection, Late Post-Injection<br>p=0.007
**Fig. 2c** Proportion of tuned units ketamine-WM sessions compared between injection periods	17<br>n=51, selectivity proportions combined (17 per epoch)	Analysis of variance with post hoc testing<br><br>Chi-Square Test	Pre-Injection, *mean*=11.36, *median*=10.93<br>Early Post-Injection, *mean*=6.23, *median*=5.20<br>Late Post-Injection, *mean*=9.62, *median*=7.79<br><br>F=8.73, p=0.0002, df=2,303<br>Post Hoc<br>Pre-Injection, Early Post-Injection<br>p=0.0001<br>Pre-Injection, Late Post-Injection<br>p=0.342<br>Early Post-Injection, Late Post-Injection<br>p=0.018<br><br>Pre-Injection- Early Post-Injection, p=0, X^2=128.67<br>Pre-Injection- Late Post-Injection, p=0.97, X^2=0.002<br>Early Post-Injection- Late Post-Injection, p=0, X^2=126.52
**Fig. 2d** Proportion of tuned units saline-WM sessions compared between injection periods	7<br>n=21, selectivity proportions combined (7 per epoch)	One-way analysis of variance<br><br>Chi-Square Test	Pre-Injection, *mean*=11.7, *median*=13.5<br>Early Post-Injection, *mean*=10.04, *median*=10.26<br>Late Post-Injection, *mean*=8.04, *median*=8.39<br><br>F=1.93, p=0.1498, df=2,123<br><br>Pre-Injection- Early Post-Injection, p=0.231, X^2=1.44

			Pre-Injection- Late Post-Injection, *p*=5.26e-07, X^2=25.17<br>Early Post-Injection- Late Post-Injection, *p*=1.3e-04, X^2=14.65
**Fig. 2f**<br>Change in slope magnitude between injection periods	17	Kruskal-Wallis one-way analysis of variance with post hoc testing	**Ketamine**<br>Pre-Injection, *mean*=-0.411, *median*=-0.413<br>Early Post-Injection, *mean*=-0.286, *median*=-0.298<br>Late Post-Injection, *mean*=-0.315, *median*=-0.306<br><br>*H*=13.48, *p*=0.0012, *df*=2,48<br><br>Post Hoc<br>Pre-Injection, Early Post-Injection, *p*=0.001<br>Pre-Injection, Late Post-Injection, *p*=0.017<br>Early Post-Injection, Late Post-Injection, *p*=0.741<br><br>**Saline**<br>*H*=5.7, *p*= 0.058, *df*=2,18<br><br>Post Hoc<br>Pre-Injection, Early Post-Injection, *p*=0.097<br>Pre-Injection, Late Post-Injection, *p*=0.097<br>Early Post-Injection, Late Post-Injection, *p*=1
**Fig. 3a**<br>SVM decoding accuracy ketamine-WM compared between injection periods	16	Kruskal-Wallis one-way analysis of variance with post hoc testing	**Cue:** *df=2*<br># of Neurons *p* *H* pre-post, *p* *CI*- pre CI-post<br>1 0.013 8.76 0.012 53.4, 59.5 46.7, 53.2<br>2 0.008 9.74 0.007 64.1, 75.9 52.2, 62.7<br>3 0.008 9.61 0.008 68.0, 80.6 55.5, 66.9<br>4 0.015 8.37 0.016 70.1, 83.3 57.9, 70.6<br>5 0.016 8.25 0.018 72.0, 85.3 59.2, 72.6<br>6 0.024 7.42 0.023 73.1, 86.9 60.3, 74.0<br>7 0.046 6.15 0.039 73.1, 87.5 61.4, 75.4<br>8 0.050 5.99 0.042 73.6, 88.0 62.0, 76.3<br>9 0.052 5.91 0.046 73.8, 88.8 62.7, 77.2<br>10 0.049 6.03 0.043 74.1, 89.5 63.0, 77.6<br>11 0.069 5.36 0.062 74.9, 89.9 63.3, 78.0<br>12 0.075 5.17 0.067 74.5, 90.1 63.4, 78.4<br>13 0.069 5.36 0.062 74.7, 90.5 63.6, 78.6<br>14 0.067 5.4 0.060 74.6, 90.8 63.7, 78.7<br>15 0.108 4.45 0.099 74.2, 90.6 63.8, 78.9<br>16 0.076 5.15 0.072 75.1, 91.1 64.0, 79.0<br><br>**Delay:** *df=2*<br># of Neurons *p* *H* pre-post, *p* *CI*-pre *CI*-post<br>1 0.002 12.78 0.002 54.7, 60.2 45.6, 51.4

2	0.004	10.86	0.006	66.9, 79.2	51.6, 62.4
3	0.008	9.79	0.011	70.5, 83.0	55.0, 67.3
4	0.009	9.43	0.016	73.1, 85.5	57.7, 70.1
5	0.013	8.63	0.023	74.0, 87.0	58.9, 71.7
6	0.006	10.21	0.013	75.5, 88.5	59.5, 72.4
7	0.004	10.91	0.012	76.4, 89.7	60.5, 73.4
8	0.006	10.21	0.018	76.9, 90.3	61.7, 74.5
9	0.006	10.34	0.017	77.5, 90.7	61.7, 75.1
10	0.004	11.06	0.010	78.5, 91.1	61.6, 75.5
11	0.002	12.71	0.006	79.7, 91.9	62.0. 75.9
12	0.003	11.35	0.012	79.2, 92.0	62.3, 76.3
13	0.004	11.08	0.015	79.4, 92.1	62.4, 76.6
14	0.004	11.07	0.013	79.1, 92.1	62.3, 76.6
15	0.004	11.07	0.014	79.2, 92.4	62.5, 77.0
16	0.004	11.26	0.015	79.4, 92.3	62.4, 77.1

Response: *df=2*

# of Neurons	*p*	*H*	pre-post, *p*	*CI*-pre	*CI*-post
1	0.007	10.03	0.007	53.6, 60.7	46.6, 52.5
2	0.014	8.48	0.010	65.4, 79.5	53.1, 63.3
3	0.010	9.32	0.007	70.3, 84.1	55.4, 67.0
4	0.007	9.94	0.005	72.8, 86.3	56.8, 68.9
5	0.009	9.42	0.007	74.0, 87.8	58.0, 71.0
6	0.009	9.46	0.007	75.8, 88.6	58.5, 72.2
7	0.006	10.18	0.006	77.5, 89.5	59.1, 72.9
8	0.011	9.08	0.013	78.1, 89.9	60.0, 74,1
9	0.011	8.96	0.014	78.7, 90.6	60.5, 74.9
10	0.010	9.15	0.014	79.5, 90.8	61.0, 75.4
11	0.010	9.25	0.014	79.5, 91.1	61.4, 75.7

			12 0.010 9.3 0.016 79.3, 91.3 61.7, 75.9<br>13 0.013 8.68 0.020 78.8, 91.3 62.0, 75.9<br>14 0.014 8.54 0.018 78.9, 91.3 61.9, 75.8<br>15 0.013 8.75 0.017 78.9, 91.4 62.0, 76.0<br>16 0.018 8 0.023 78.8, 91.6 62.1, 76.3
**Fig. 3b** SVM decoding accuracy saline-WM compared between injection periods	7	Kruskal-Wallis one-way analysis of variance	Ensemble of 16 neurons:<br><br>**Cue:**<br>Pre-Injection, *mean*=82.6, *median*=89.7<br>Early Post-Injection, *mean*=74.4, *median*=91.0<br>Late Post-Injection, *mean*=76.0, *median*=88.6<br><br>*H*=0.54, *p*=0.763, *df*=2,18<br><br>**Delay:**<br>Pre-Injection, *mean*=83.6, *median*=92.5<br>Early Post-Injection, *mean*=77.3, *median*=90.9<br>Late Post-Injection, *mean*=73.6, *median*=89.2<br><br>*H*=1.12, *p*=0.571, *df*=2,18<br><br>**Response:**<br>Pre-Injection, *mean*=83.9, *median*=94.1<br>Early Post-Injection, *mean*=79.3, *median*=91.1<br>Late Post-Injection, *mean*=76.9, *median*=85.9<br><br>*H*=1.36, *p*=0.507, *df*=2,18<br><br>All other neuron ensemble sizes, *p*>0.05
**Fig. 3c** LDA decoding accuracy ketamine-WM compared between injection periods	17	Kruskal-Wallis one-way analysis of variance with post hoc testing	Pre-Injection, *mean*=78.0, *median*=78.9<br>Early Post-Injection, *mean*=66.0, *median*=63.6<br>Late Post-Injection, *mean*=69.0, *median*=64.7<br><br>*H*=13.37, *p*=0.001, *df*=2,48<br>Post Hoc<br>Pre-Injection, Early Post-Injection, *p*=0.001<br>Pre-Injection, Late Post-Injection, *p*=0.038<br>Early Post-Injection, Late Post-Injection, *p*=0.495

**Fig. 3c**<br>LDA decoding accuracy ketamine-WM compared to chance (50%)	17	One sample t-test, 2-tailed	Pre-Injection, T=13.10, p=5.70e-10, df=16<br>Early Post-Injection, T=9.22, p=8.42e-08, df=16<br>Last Post-Injection, T=10.58, p=1.24e-08, df=16
**Fig. 3d**<br>Theoretical decoding accuracy ketamine-WM compared between injection periods	17	Kruskal-Wallis one-way analysis of variance with post hoc testing	Pre-Injection, *mean*=80.5, *median*=82.9<br>Early Post-Injection, *mean*=67.4, *median*=65.6<br>Late Post-Injection, *mean*=69.8, *median*=66<br><br>H=17.96, p=0.0001, df=2,48<br>Post Hoc<br>Pre-Injection, Early Post-Injection, p=0.0002<br>Pre-Injection, Late Post-Injection, p=0.004<br>Early Post-Injection, Late Post-Injection, p=0.684
**Fig. 3e**<br>Population signal compared between injection periods	Ketamine-WM<br>17<br><br>Saline-WM<br>7	Kruskal-Wallis one-way analysis of variance with post hoc testing	**Ketamine-WM**<br>Pre-Injection, *mean*=4.8, *median*=5.1<br>Early Post-Injection, *mean*=2.8, *median*=2.6<br>Late Post-Injection, *mean*=3.8, *median*=3.5<br><br>H=8.13, p=0.017, df=2,48<br>Post Hoc<br>Pre-Injection, Early Post-Injection, p=0.012<br>Pre-Injection, Late Post-Injection, p=0.374<br>Early Post-Injection, Late Post-Injection, p=0.286<br><br>**Saline-WM**<br>Pre-Injection, *mean*=5.2, *median*=6.3<br>Early Post-Injection, *mean*=4.0, *median*=5.0<br>Late Post-Injection, *mean*=3.3, *median*=3.9<br><br>H=3.44, p=0.179, df=2,18
**Fig. 3f**<br>Projected precision ketamine-WM compared between	Ketamine-WM<br>17<br><br>Saline-WM<br>7	Kruskal-Wallis one-way analysis of variance with post hoc testing	**Ketamine-WM**<br>Pre-Injection, *mean*=0.49, *median*=0.44<br>Early Post-Injection, *mean*=0.43, *median*=0.39<br>Late Post-Injection, *mean*=0.38, *median*=0.34<br><br>H=8.2, p=0.017, df=2,48

injection periods			Post Hoc<br>Pre-Injection, Early Post-Injection, p=0.380<br>Pre-Injection, Late Post-Injection, p=0.012<br>Early Post-Injection, Late Post-Injection, p=0.275<br><br>**Saline-WM**<br>Pre-Injection, *mean*=0.44, *median*=0.42<br>Early Post-Injection, *mean*=0.42, *median*=0.42<br>Late Post-Injection, *mean*=0.42, *median*=0.37<br><br>H=0.36, p=0.834, df=2,18
**Fig. 4d**<br>Change in FR between injection periods for narrow spiking neurons	17<br>n=13 (arrays per session containing selective neurons)	Wilcoxon signed-ranks test, 1-tailed	**Preferred Location,**<br>Pre-Injection, *mean*=0.45, *median*=0.47<br>Post-Injection, *mean*=0.28, *median*=0.21<br><br>Z=1.66, p=0.049<br><br>**Least-Preferred Location,**<br>Pre-Injection, *mean*=0.21, *median*=0.13<br>Post-Injection, *mean*=0.24, *median*=0.09<br><br>Z=-0.116, p=0.546
**Fig. 4f**<br>Change in FR between injection periods for broad spiking neurons	17<br>n=27 (arrays per session containing selective neurons)	Wilcoxon signed-ranks test, 1-tailed	**Preferred Location,**<br>Pre-Injection, *mean*=0.43, *median*=0.43<br>Post-Injection, *mean*=0.42, *median*=0.43<br><br>Z=0.383, p=0.649<br><br>**Least-Preferred Location,**<br>Pre-Injection, *mean*=0.23, *median*=0.25<br>Post-Injection, *mean*=0.34, *median*=0.36<br><br>Z=-2.50, p=0.006
**Fig. 5b**<br>Percent of fixation time on target	Ketamine<br>18<br><br>Saline<br>7	Two-way analysis of variance with interaction	**Ketamine:**<br>Pre-Injection, *mean*=6.97, *median*=3.64<br>Early Post-Injection, *mean*=16.92, *median*=13.07<br>Late Post-Injection, *mean*=6.98, *median*=4.25<br><br>**Saline:**<br>Pre-Injection, *mean*=7.28, *median*=1.85

			Early Post-Injection, *mean*=6.65, *median*=2.15<br>Late Post-Injection, *mean*=5.73, *median*=2.86<br><br>Drug: *F*=1.73, *p*=0.193, *df*=1,69<br>Injection Period: *F*=1.42, *p*=0.248, *df*=2,69<br>Interaction: *F*=1.35, *p*=0.267, *df*=2,69
**Fig. 5c**<br>Eye data SVM decoding compared between injection periods	16	Kruskal-Wallis one-way analysis of variance	**Cue:**<br>Pre-Injection, *mean*=53.9, *median*=57.6<br>Early Post-Injection, *mean*=52.2, *median*=52.0<br>Late Post-Injection, *mean*=57.7, *median*=59.3<br><br>*H*=4.01, *p*=0.135, *df*=2,45<br><br>**Delay:**<br>Pre-Injection, *mean*=47.8, *median*=47.8<br>Early Post-Injection, *mean*=46.5, *median*=46.7<br>Late Post-Injection, *mean*=52.1, *median*=51.8<br><br>*H*=4.59, *p*=0.101, *df*=2,45
**Fig. 5c**<br>Eye data SVM decoding accuracy compared between cue and delay epochs	16<br>*n*=48, data combined between injection periods	Wilcoxon signed-ranks test, 2-tailed	*Z*=3.18, *p*=0.002
**Fig. 5d**<br>SVM decoding accuracy for eye data compared to decoding accuracy for neural ensembles	16	Kruskal-Wallis one-way analysis of variance	**Cue:**<br>Eye data<br>*mean*=53.9, *median*=57.6<br>Neural data<br>*mean*=83.6, *median*=91<br><br>*H*=14.78, *p*=0.0001, *df*=1,30<br><br>**Delay:**<br>Eye data<br>*mean*=47.8, *median*=47.8<br>Neural data<br>*mean*=86.2, *median*=91.9

			H=22.91, p=1.69e-06, df=1,30
**Fig. 5d**<br>Eye data decoding accuracy compared to chance (33%)	16	One sample t-test, 2-tailed	**Cue:** T=8.38, p=4.82e-07, df=15<br><br>**Delay:** T=8.53, p=3.87e-07, df=15
**Extended Data 1a, b**<br>Task performance (percent correct) compared between injection periods	NHP B<br>Ketamine-WM<br>9<br>Saline-WM<br>4<br><br>NHP T<br>Ketamine-WM<br>9<br>Saline-WM<br>3	Repeated measures analysis of variance with post hoc pairwise comparisons<br><br><br><br><br><br><br><br><br><br><br><br><br><br><br><br><br><br><br>Two-way analysis of variance	**NHP B**<br>**Ketamine-WM:**<br>Pre-Injection, *mean*=89, *median*=90<br>Early Post-Injection, *mean*=39, *median*=43<br>Late Post-Injection, *mean*=80, *median*=83<br><br>F=22.49, p<0.0001, df=2,16<br>Post Hoc<br>Pre-Injection, Early Post-Injection, p<0.0001<br>Pre-Injection, Late Post-Injection, p=0.442<br>Early Post-Injection, Late Post-Injection, p=0.0003<br><br>**Saline-WM:**<br>Pre-Injection, *mean*=91, *median*=90<br>Early Post-Injection, *mean*=93, *median*=92<br>Late Post-Injection, *mean*=84, *median*=84<br><br>F=3.88, p=0.083, df=2,6<br><br>Drug: F=12.95, p=0.001, df=1,33<br>Injection Period: F=6.78, p=0.0034, df=2,33<br>Interaction: F=9.57, p=0.0005, df=2,33<br><br>Ket pre-injection, Sal pre-injection, p=1<br>Ket pre-injection, Ket early post-injection, p=0<br>Ket pre-injection, Sal early post-injection, p=0.999<br>Ket pre-injection, Ket late post-injection, p=0.763<br>Ket pre-injection, Sal late post-injection, p=0.988<br>Sal pre-injection, Ket early post-injection, p=0.0001<br>Sal pre-injection, Sal early post-injection, p=1<br>Sal pre-injection, Ket late post-injection, p=0.827<br>Sal pre-injection, Sal late post-injection, p=0.984

			Ket early post-injection, Sal early post-injection, p=0<br>Ket early post-injection, Ket late post-injection, p=0.0001<br>Ket early post-injection, Sal late post-injection, p=0.0006<br>Sal early post-injection, Ket late post-injection, p=0.735<br>Sal early post-injection, Sal late post-injection, p=0.962<br>Ket late post-injection, Sal late post-injection, p=0.998
		Repeated measures analysis of variance with post hoc pairwise comparisons	**NHP T**<br>**Ketamine-WM:**<br>Pre-Injection, *mean*=54, *median*=60<br>Early Post-Injection, *mean*=30, *median*=24<br>Late Post-Injection, *mean*=47, *median*=56<br><br>F=6.19, p=0.010, df=2,16<br>Post Hoc<br>Pre-Injection, Early Post-Injection, p=0.0099<br>Pre-Injection, Late Post-Injection, p=0.662<br>Early Post-Injection, Late Post-Injection, p=0.057<br><br>**Saline-WM:**<br>Pre-Injection, *mean*=65, *median*=70<br>Early Post-Injection, *mean*=55, *median*=56<br>Late Post-Injection, *mean*=42, *median*=57<br><br>F=1.55, p=0.318, df=2,4
		Two-way analysis of variance	Drug: F=2.76, p=0.107, df=1,30<br>Injection Period: F=2.99, p=0.066, df=2,30<br>Interaction: F=2.15, p=0.135, df=2,30<br><br>Ket pre-injection, Sal pre-injection, p=0.894<br>Ket pre-injection, Ket early post-injection, p=0.037<br>Ket pre-injection, Sal early post-injection, p=1<br>Ket pre-injection, Ket late post-injection, p=0.961<br>Ket pre-injection, Sal late post-injection, p=0.864<br>Sal pre-injection, Ket early post-injection, p=0.027<br>Sal pre-injection, Sal early post-injection, p=0.972<br>Sal pre-injection, Ket late post-injection, p=0.581<br>Sal pre-injection, Sal late post-injection, p=0.487

			Ket early post-injection, Sal early post-injection, p=0.198<br>Ket early post-injection, Ket late post-injection, p=0.205<br>Ket early post-injection, Sal late post-injection, p=0.873<br>Sal early post-injection, Ket late post-injection, p=0.980<br>Sal early post-injection, Sal late post-injection, p=0.904<br>Ket late post-injection, Sal late post-injection, p=0.993
**Extended Data 1c, d**<br>Response time compared between injection periods	NHP B<br>9<br>n=64<br><br>NHP T<br>9<br>n=62	Repeated measures analysis of variance with post hoc pairwise comparisons	**NHP B:**<br>Pre-Injection, *mean*=2.6, *median*=2.4<br>Early Post-Injection, *mean*=3.5, *median*=3.0<br>Late Post-Injection, *mean*=2.7, *median*=2.5<br><br>F=19.73, p=3.50e-08, df=2,126<br>Post Hoc<br>Pre-Injection, Early Post-Injection, $p<0.0001$<br>Pre-Injection, Late Post-Injection, p=0.553<br>Early Post-Injection, Late Post-Injection, $p<0.0001$<br><br>**NHP T:**<br>Pre-Injection, *mean*=2.7, *median*=2.3<br>Early Post-Injection, *mean*=2.9, *median*=2.8<br>Late Post-Injection, *mean*=2.8, *median*=2.4<br><br>F=1.5, p=0.226, df=2,122
**Extended Data 2a, b**<br>Proportion of tuned units ketamine-WM compared between injection periods	NHP B<br>9<br>n=27<br><br>NHP T<br>8<br>n=24	Analysis of variance with post hoc testing<br><br>Chi-Square Test<br><br>Analysis of variance with	**NHP B:**<br><br>F=9.9, $p<0.0001$, df=2,159<br>Post Hoc<br>Pre-Injection, Early Post-Injection, p=0.0001<br>Pre-Injection, Late Post-Injection, p=0.820<br>Early Post-Injection, Late Post-Injection, p=0.0013<br><br>Pre-Injection, Early Post-Injection, p=0, X^2=133.83<br>Pre-Injection, Late Post-Injection, p=0.62, X^2=0.25<br>Early Post-Injection, Late Post-Injection, p=0, X^2=143.7<br><br>**NHP T:**<br>F=3.96, p=0.021, df=2,141<br>Post Hoc<br>Pre-Injection, Early Post-Injection, p=0.019

		post hoc testing<br><br><br>Chi-Square Test	Pre-Injection, Late Post-Injection, p=0.108<br>Early Post-Injection, Late Post-Injection, p=0.769<br><br>Pre-Injection, Early Post-Injection, p=0.14, X^2=2.22<br>Pre-Injection, Late Post-Injection, p=0.105, X^2=2.63<br>Early Post-Injection, Late Post-Injection, p=0.89, X^2=0.018
**Extended Data 2c** Overall change in FR between injection periods	17<br>Cue, n=604 neurons<br>Delay, n=475 neurons<br>Response, n=671 neurons	One-way analysis of variance	**Cue:**<br>Pre-Injection, *mean*=8.7, *median*=6.1<br>Post-Injection, *mean*=9.7, *median*=6.5<br><br>F=3.28, p=0.073, df=1,1206<br><br>**Delay:**<br>Pre-Injection, *mean*=8.2, *median*=5.6<br>Post-Injection, *mean*=8.8, *median*=5.7<br><br>F=1.21, p=0.272, df=1,948<br><br>**Response:**<br>Pre-Injection, *mean*=8.4, *median*=5.6<br>Post-Injection, *mean*=9.0, *median*=5.7<br><br>F=1.56, p=0.212, df=1,1340
**Extended Data 3c, d** SVM decoding accuracy ketamine-WM compared between injection periods	NHP T<br>7<br>NHP B<br>9	Kruskal-Wallis one-way analysis of variance with post hoc testing	**NHP T: Ensembles of 16 neurons**<br>**Cue:** H=1.9, p=0.387, df=2,18<br><br>**Delay:** H=8.22, p=0.016, df=2,18<br>Post Hoc<br>Pre-Injection, Early Post-Injection, p=0.021<br>Pre-Injection, Late Post-Injection, p=0.065<br>Early Post-Injection, Late Post-Injection, p=0.903<br><br>**Response:** H=6.24, p=0.044, df=2,18<br>Post Hoc<br>Pre-Injection and Early Post-Injection, p=0.033<br>Pre-Injection and Late Post-Injection, p=0.424<br>Early Post-Injection and Late Post-Injection, p=0.424

			**NHP B: Ensembles of 16 neurons**<br>**Cue:** *H*=9.76, *p*=0.008, *df*=2,24<br>Post Hoc<br>Pre-Injection and Early Post-Injection, *p*=0.008<br>Pre-Injection and Late Post-Injection, *p*=0.062<br>Early Post-Injection and Late Post-Injection, *p*=0.738<br><br>**Delay:** *H*=12.67, *p*=0.002, *df*=2,24<br>Post Hoc<br>Pre-Injection, Early Post-Injection, *p*=0.009<br>Pre-Injection, Late Post-Injection, *p*=0.004<br>Early Post-Injection, Late Post-Injection, *p*=0.961<br><br>**Response:** *H*=6.43, *p*=0.040, *df*=2,24<br>Post Hoc<br>Pre-Injection, Early Post-Injection, *p*=0.054<br>Pre-Injection, Late Post-Injection, *p*=0.100<br>Early Post-Injection, Late Post-Injection, *p*=0.961
**Extended Data 3c, d** SVM decoding accuracy ketamine-WM compared to chance (33%)	NHP T<br>7<br>NHP B<br>9	One sample t-test, 2-tailed	**NHP T: Ensembles of 16 neurons**<br>**Cue:**<br>Pre: *T*=5.5, *p*=0.002, *df*=6<br>Early-Post: *T*=14.3, *p*=7.39e-06, *df*=6<br>Late-Post: *T*=8.32, *p*=0.0002, *df*=6<br><br>**Delay:**<br>Pre: *T*=8.42, *p*=0.0002, *df*=6<br>Early-Post: *T*=7.9, *p*=0.0002, *df*=6<br>Late-Post: *T*=14.8, *p*=5.97e-06, *df*=6<br><br>**Response:**<br>Pre: *T*=8.65, *p*=0.0001, *df*=6<br>Early-Post: *T*=8.04, *p*=0.0002, *df*=6<br>Late-Post: *T*=12.18, *p*=1.86e-05, *df*=6<br><br>**NHP B: Ensembles of 16 neurons**<br>**Cue:**<br>Pre: *T*=74.78, *p*=1.14e-12, *df*=8<br>Early-Post: *T*=17.15, *p*=1.36e-07, *df*=8<br>Late-Post: *T*=17.05, *p*=1.42e-07, *df*=8

			**Delay:**<br>Pre: *T*=53.21, *p*=1.73e-11, *df*=8<br>Early-Post: *T*=11.88, *p*=2.32e-06, *df*=8<br>Late-Post: *T*=8.21, *p*=3.62e-05, *df*=8<br><br>**Response:**<br>Pre: *T*=37.13, *p*=3.04e-10, *df*=8<br>Early-Post: *T*=9.81, *p*=9.79e-06, *df*=8<br>Late-Post: *T*=7.67, *p*=5.90e-05, *df*=8
**Extended Data 3e** Decoding accuracy compared to shuffled results	16	Kruskal-Wallis one-way analysis of variance	Pre-Injection:<br>*H*=71.26, *p*=3.13e-17, *df*=1,94<br><br>Early Post-Injection:<br>*H*=71.26, *p*=3.13e-17, *df*=1,94<br><br>Late Post-Injection:<br>*H*=71.26, *p*=3.13e-17, *df*=1,94
**Extended Data 4a** Correct trial SVM decoding accuracy compared between injection periods	Pre-injection 13<br><br>Early Post-Injection 12<br><br>Late Post-Injection 16	Kruskal-Wallis one-way analysis of variance with post hoc testing	**Cue:**<br>Pre-Injection, *mean*=94.5, *median*=95.8<br>Early Post-Injection, *mean*=76.9, *median*=71.4<br>Late Post-Injection, *mean*=82.4, *median*=88.3<br><br>*H*=11.11, *p*=0.004, *df*=2,38<br>Post Hoc<br>Pre-Injection, Early Post-Injection, *p*=0.004<br>Pre-Injection, Late Post-Injection, *p*=0.031<br>Early Post-Injection, Late Post-Injection, *p*=0.671<br><br>**Delay:**<br>Pre-Injection, *mean*=93.4, *median*=95.2<br>Early Post-Injection, *mean*=80.5, *median*=79.5<br>Late Post-Injection, *mean*=86.6, *median*=90.9<br><br>*H*=8.35, *p*=0.015, *df*=2,38<br>Post Hoc<br>Pre-Injection and Early Post-Injection, *p*=0.012<br>Pre-Injection and Late Post-Injection, *p*=0.139<br>Early Post-Injection and Late Post-Injection, *p*=0.499

			**Response:**<br>Pre-Injection, *mean*=95.8, *median*=96.3<br>Early Post-Injection, *mean*=87.6, *median*=92.9<br>Late Post-Injection, *mean*=90.4, *median*=94<br><br>H=5.5, p=0.064, df=2,38<br>Post Hoc<br>Pre-Injection and Early Post-Injection, p=0.062<br>Pre-Injection and Late Post-Injection, p=0.201<br>Early Post-Injection and Late Post-Injection, p=0.768
**Extended Data 4a** Correct trial SVM decoding accuracy compared to chance (33%)	Pre-injection 13<br><br>Early Post-Injection 12<br><br>Late Post-Injection 16	One sample t-test, 2-tailed	**Cue:**<br>Pre: T=40.61, p=3.21e-14, df=12<br>Early-Post: T=10.19, p=6.1e-07, df=11<br>Late-Post: T=13.38, p=9.65e-10, df=15<br><br>**Delay:**<br>Pre: T=28.01, p=2.66e-12, df=12<br>Early-Post: T=12.88, p=5.6e-08, df=11<br>Late-Post: T=18.84, p=7.46e-12, df=15<br><br>**Response:**<br>Pre: T=52.63, p=1.46e-15, df=12<br>Early-Post: T=15.05, p=1.1e-08, df=11<br>Late-Post: T=24.81, p=1.36e-13, df=15
**Extended Data 4b** SVM decoding accuracy compared between correct and all trials	Pre-injection 13<br><br>Early Post-Injection 12<br><br>Late Post-Injection 16	Wilcoxon signed-ranks test, 1-tailed	**Pre-Injection Period:**<br>Cue: Z=1.38, p=0.083<br>Delay: Z=0.719, p=0.236<br>Response: Z=1.44, p=0.075<br><br>**Early Post-Injection Period:**<br>Cue: Z=1.53, p=0.063<br>Delay: Z=1.76, p=0.039<br>Response: Z=2.80, p=0.003<br><br>**Late Post-Injection Period**<br>Cue: Z=1.52, p=0.063<br>Delay: Z=3.07, p=0.001

			Response: *Z*=3.41, *p*=0.0003
**Extended Data 5b** LDA decoding accuracy compared to theoretical decoding accuracy	17<br>*n*=51 (values combined between trial epochs for pre-injection period)	Kruskal-Wallis one-way analysis of variance	*H*=1.87, *p*=0.171, *df*=1,100
**Extended Data 5c, e** LDA decoding accuracy ketamine-WM compared between injection periods	NHP B<br>9<br><br>NHP T<br>8	Kruskal-Wallis one-way analysis of variance with post hoc testing	**NHP B:**<br>Pre-Injection, *mean*=84.3, *median*=85.6<br>Early Post-Injection, *mean*=69.8, *median*=66.5<br>Late Post-Injection, *mean*=73.4, *median*=77.7<br><br>*H*=13.24, *p*=0.001, *df*=2,24<br>Pre-Injection, Early Post-Injection, *p*=0.002<br>Pre-Injection, Late Post-Injection, *p*=0.021<br>Early Post-Injection, Late Post-Injection, *p*=0.702<br><br>**NHP T:**<br>Pre-Injection, *mean*=70.8, *median*=70.9<br>Early Post-Injection, *mean*=61.6, *median*=62.0<br>Late Post-Injection, *mean*=64.0, *median*=63.7<br><br>*H*=8.42, *p*=0.015, *df*=2,21<br>Post Hoc<br>Pre-Injection, Early Post-Injection, *p*=0.011<br>Pre-Injection, Late Post-Injection, *p*=0.265<br>Early Post-Injection, Late Post-Injection, *p*=0.371
**Extended Data 5c, e** LDA decoding accuracy ketamine-WM compared to chance (50%)	NHP B<br>9<br><br>NHP T<br>8	One sample t-test, 2-tailed	**NHP B**<br>Pre-Injection: *T*=25.15, *p*=6.68e-09, *df*=8<br>Early Post-Injection: *T*=7.63, *p*=6.12e-05, *df*=8<br>Late Post-Injection: *T*=9.11, *p*=1.70e-05, *df*=8<br><br>**NHP T**<br>Pre-Injection: *T*=8.49, *p*=6.21e-05, *df*=7<br>Early Post-Injection: *T*=12.75, *p*=4.23e-06, *df*=7<br>Late Post-Injection: *T*=18.28, *p*=3.63e-07, *df*=7

**Extended Data 5d, f** Theoretical decoding accuracy ketamine-WM compared between injection periods	NHP B<br>9<br><br>NHP T<br>8	Kruskal-Wallis one-way analysis of variance with post hoc testing	**NHP B:**<br>Pre-Injection, *mean*=85.1, *median*=85.9<br>Early Post-Injection, *mean*=71.3, *median*=67.9<br>Late Post-Injection, *mean*=73.3, *median*=76.9<br><br>H=13.36, p=0.001, df=2,24<br>Post Hoc<br>Pre-Injection, Early Post-Injection, p=0.004<br>Pre-Injection, Late Post-Injection, p=0.006<br>Early Post-Injection, Late Post-Injection, p=0.988<br><br>**NHP T:**<br>Pre-Injection, *mean*=75.3, *median*=77.4<br>Early Post-Injection, *mean*=63.0, *median*=62.8<br>Late Post-Injection, *mean*=65.9, *median*=65.2<br><br>H=11.54, p=0.003, df=2,21<br>Post Hoc<br>Pre-Injection, Early Post-Injection, p=0.002<br>Pre-Injection, Late Post-Injection, p=0.169<br>Early Post-Injection, Late Post-Injection, p=0.250
**Extended Data 5g** LDA decoding accuracy saline-WM compared between injection periods	7	Kruskal-Wallis one-way analysis of variance	Pre-Injection, *mean*=77.0, *median*=79.5<br>Early Post-Injection, *mean*=72.9, *median*=77.3<br>Late Post-Injection, *mean*=68.9, *median*=73.5<br><br>H=2.43, p=0.297, df=2,18
**Extended Data 5h** Theoretical decoding accuracy saline-WM compared between injection periods	7	Kruskal-Wallis one-way analysis of variance	Pre-Injection, *mean*=79.6, *median*=81.2<br>Early Post-Injection, *mean*=73.4, *median*=77.1<br>Late Post-Injection, *mean*=70.2, *median*=73.1<br><br>H=3.95, p=0.139, df=2,18

**Extended Data 5i, k** Population signal ketamine-WM compared between injection periods	NHP B<br>9<br><br>NHP T<br>8	Kruskal-Wallis one-way analysis of variance with post hoc testing	**NHP B:**<br>Pre-Injection, *mean*=6.4, *median*=6.3<br>Early Post-Injection, *mean*=3.8, *median*=3.9<br>Late Post-Injection, *mean*=5.2, *median*=5.4<br><br>*H*=12.91, *p*=0.001, *df*=2,24<br>Post Hoc<br>Pre-Injection, Early Post-Injection, *p*=0.001<br>Pre-Injection, Late Post-Injection, *p*=0.186<br>Early Post-Injection, Late Post-Injection, *p*=0.156<br><br>**NHP T:**<br>Pre-Injection, *mean*=2.9, *median*=2.5<br>Early Post-Injection, *mean*=1.7, *median*=1.6<br>Late Post-Injection, *mean*=2.1, *median*=2.0<br><br>*H*=4.82, *p*=0.090, *df*=2,21<br>Post Hoc<br>Pre-Injection, Early Post-Injection, *p*=0.073<br>Pre-Injection, Late Post-Injection, *p*=0.583<br>Early Post-Injection, Late Post-Injection, *p*=0.452
**Extended Data 5j, l** Projected precision ketamine-WM compared between injection periods	NHP B<br>9<br><br>NHP T<br>8	Kruskal-Wallis one-way analysis of variance with post hoc testing	**NHP B:**<br>Pre-Injection, *mean*=0.40, *median*=0.40<br>Early Post-Injection, *mean*=0.39, *median*=0.37<br>Late Post-Injection, *mean*=0.30, *median*=0.32<br><br>*H*=14.51, *p*=0.0007, *df*=2,24<br>Post Hoc<br>Pre-Injection, Early Post-Injection, *p*=0.646<br>Pre-Injection, Late Post-Injection, *p*=0.0008<br>Early Post-Injection, Late Post-Injection, *p*=0.016<br><br>**NHP T:**<br>Pre-Injection, *mean*=0.60, *median*=0.58<br>Early Post-Injection, *mean*=0.48, *median*=0.52<br>Late Post-Injection, *mean*=0.47, *median*=0.48<br><br>*H*=3.86, *p*=0.145, *df*=2,21<br>Post Hoc<br>Pre-Injection, Early Post-Injection, *p*=0.235<br>Pre-Injection, Late Post-Injection, *p*=0.181<br>Early Post-Injection, Late Post-Injection, *p*=0.989

**Extended Data 6a, b**<br>Change in FR for narrow and broad spiking neurons saline-WM	7<br>Narrow, *n*=5 (arrays per session containing selective neurons)<br>Broad, *n*=11	Wilcoxon signed-ranks test, 1-sided	**Narrow:**<br>**Preferred Location,**<br>Pre-Injection, *mean*=0.43, *median*=0.47<br>Post-Injection, *mean*=0.42, *median*=0.48<br><br>*p*=0.500<br><br><br>**Least-Preferred Location,**<br>Pre-Injection, *mean*=0.32, *median*=0.35<br>Post-Injection, *mean*=0.26, *median*=0.33<br><br>*p*=0.500<br><br><br>**Broad:**<br>**Preferred Location,**<br>Pre-Injection, *mean*=0.52, *median*=0.51<br>Post-Injection, *mean*=0.42, *median*=0.45<br><br>*p*=0.803<br><br><br>**Least-Preferred Location,**<br>Pre-Injection, *mean*=0.29, *median*=0.29<br>Post-Injection, *mean*=0.32, *median*=0.28<br><br>*p*=0.422
**Extended Data 6c**<br>Narrow spiking ranked target location responses	Narrow, *n*=13	Wilcoxon signed-ranks test, 1-sided	 **Rank 1**<br>Pre-Injection, *mean*=0.343, *median*=0.363<br>Post-Injection, *mean*=0.212, *median*=0.161<br>*Z=1.66, p*=0.049<br><br>**Rank 2**<br>Pre-Injection, *mean*=0.302, *median*=0.383<br>Post-Injection, *mean*=0.245, *median*=0.255<br>*Z=0.843, p*=0.120<br><br>**Rank 3**<br>Pre-Injection, *mean*=0.264, *median*=0.323<br>Post-Injection, *mean*=0.278, *median*=0.282<br>*Z*=-0.217, *p*=0.586<br><br>**Rank 4**

			Pre-Injection, *mean*=0.246, *median*=0.224<br>Post-Injection, *mean*=0.254, *median*=0.164<br>*Z=0.232, p*=0.592<br><br>**Rank 5**<br>Pre-Injection, *mean*=0.219, *median*=0.242<br>Post-Injection, *mean*=0.230, *median*=0.234<br>*Z=0.027, p*=0.511<br><br>**Rank 6**<br>Pre-Injection, *mean*=0.210, *median*=0.213<br>Post-Injection, *mean*=0.246, *median*=0.194<br>*Z=-0.299, p*=0.382<br><br>**Rank 7**<br>Pre-Injection, *mean*=0.196, *median*=0.147<br>Post-Injection, *mean*=0.274, *median*=0.298<br>*Z=-0.708, p*=0.240<br><br>**Rank 8**<br>Pre-Injection, *mean*=0.204, *median*=0.184<br>Post-Injection, *mean*=0.312, *median*=0.278<br>*Z=-0.380, p*=0.352<br><br>**Rank 9**<br>Pre-Injection, *mean*=0.158, *median*=0.102<br>Post-Injection, *mean*=0.182, *median*=0.072<br>*Z=0, p*=0.500
**Extended Data 6d** Broad spiking ranked target location responses	Broad, *n*=27	Wilcoxon signed-ranks test, 1-sided	**Rank 1**<br>Pre-Injection, *mean*=0.388, *median*=0.388<br>Post-Injection, *mean*=0.375, *median*=0.383<br>*Z=0.436, p*=0.332<br><br>**Rank 2**<br>Pre-Injection, *mean*=0.348, *median*=0.366<br>Post-Injection, *mean*=0.323, *median*=0.325<br>*Z=0.779, p*=0.218<br><br>**Rank 3**<br>Pre-Injection, *mean*=0.343, *median*=0.331<br>Post-Injection, *mean*=0.358, *median*=0.395<br>*Z=-0.621, p*=0.733<br><br>**Rank 4**<br>Pre-Injection, *mean*=0.320, *median*=0.312

			Post-Injection, *mean*=0.341, *median*=0.357<br>*Z=-0.349, p*=0.364<br><br>**Rank 5**<br>Pre-Injection, *mean*=0.299, *median*=0.298<br>Post-Injection, *mean*=0.350, *median*=0.358<br>*Z=-1.34, p*=0.09<br><br>**Rank 6**<br>Pre-Injection, *mean*=0.284, *median*=0.280<br>Post-Injection, *mean*=0.341, *median*=0.323<br>*Z=-1.01, p*=0.156<br><br>**Rank 7**<br>Pre-Injection, *mean*=0.242, *median*=0.249<br>Post-Injection, *mean*=0.273, *median*=0.290<br>*Z=-0.711, p*=0.238<br><br>**Rank 8**<br>Pre-Injection, *mean*=0.229, *median*=0.244<br>Post-Injection, *mean*=0.268, *median*=0.301<br>*Z=-1.318, p*=0.094<br><br>**Rank 9**<br>Pre-Injection, *mean*=0.211, *median*=0.223<br>Post-Injection, *mean*=0.299, *median*=0.321<br>*Z=-2.30, p*=0.011
**Extended Data 6i,j,k,l**<br>Change in FR for narrow and broad spiking neurons ketamine-WM	NHP T<br>8<br>Narrow, *n*=3 (arrays per session containing selective neurons)<br>Broad, *n*=9<br><br>NHP B<br>9<br>Narrow, *n*=10 (arrays per session containing selective neurons)	Wilcoxon signed-ranks test, 1-sided	**NHP T, Narrow:**<br>**Preferred Location,**<br>Pre-Injection, *mean*=0.65, *median*=0.85<br>Post-Injection, *mean*=0.09, *median*=0.12<br><br>*p*=0.200<br><br>**Least-Preferred Location,**<br>Pre-Injection, *mean*=0.05, *median*=0.04<br>Post-Injection, *mean*=0.19, *median*=0.11<br><br>*p*=0.900<br><br>**NHP T, Broad:**<br>**Preferred Location,**

	Broad, *n*=18		Pre-Injection, *mean*=0.40, *median*=0.47<br>Post-Injection, *mean*=0.38, *median*=0.39<br><br>*p*=0.697<br><br><br>**Least-Preferred Location,**<br>Pre-Injection, *mean*=0.20, *median*=0.22<br>Post-Injection, *mean*=0.34, *median*=0.33<br><br>*p*=0.081<br><br><br>**NHP B, Narrow:**<br>**Preferred Location,**<br>Pre-Injection, *mean*=0.55, *median*=0.59<br>Post-Injection, *mean*=0.36, *median*=0.36<br><br>*p*=0.039<br><br><br>**Least-Preferred Location,**<br>Pre-Injection, *mean*=0.26, *median*=0.22<br>Post-Injection, *mean*=0.31, *median*=0.33<br><br>*p*=0.552<br><br><br>**NHP B, Broad:**<br>**Preferred Location,**<br>Pre-Injection, *mean*=0.46, *median*=0.43<br>Post-Injection, *mean*=0.45, *median*=0.44<br><br>*p*=0.654<br><br><br>**Least-Preferred Location,**<br>Pre-Injection, *mean*=0.26, *median*=0.26<br>Post-Injection, *mean*=0.35, *median*=0.36<br><br>*p*=0.003
**Extended Data 7a, c** Theoretical decoding for	Broad<br>17 sessions	Kruskal-Wallis one-way analysis of variance	**Broad:**<br>Pre-Injection, *mean*=81.5, *median*=84.7<br>Post-Injection, *mean*=67.9, *median*=66.4

broad and narrow spiking neurons compared between injection periods	Narrow<br>12 sessions		*H*=13.71, *p*=0.0002, *df*=1,32<br><br>**Narrow:**<br>Pre-Injection, *mean*=79.7, *median*=77.8<br>Post-Injection, *mean*=69.1, *median*=66.1<br><br>*H*=5.07, *p*=0.024, *df*=1,22
**Extended Data 7b, d** Population signal for broad and narrow spiking neurons compared between injection periods	Broad<br>17 sessions<br><br>Narrow<br>12 sessions	Kruskal-Wallis one-way analysis of variance	**Broad:**<br>Pre-Injection, *mean*=5.13, *median*=5.36<br>Post-Injection, *mean*=3.16, *median*=2.79<br><br>*H*=7.5, *p*=0.006, *df*=1,32<br><br>**Narrow:**<br>Pre-Injection, *mean*=4.89, *median*=4.04<br>Post-Injection, *mean*=3.05, *median*=2.82<br><br>*H*=1.33, *p*=0.248, *df*=1,22
**Extended Data 9c** Decoding accuracy compared to chance (25%)	Cue<br>11<br><br>Delay<br>7	One sample t-test, 2-tailed	**Cue:**<br>*mean*=31.7, *CI*=24.3, 39.0, *median*=30.0<br>*T*=1.72, *p*=0.117, *df*=10<br><br>**Delay:**<br>*mean*=23.3, *CI*=17.6, 32.6, *median*=22.4<br>*T*=-0.48, *p*=0.646, *df*=6
**Extended Data 10a-f** Eyes on screen time compared between injection periods	Ketamine-WM<br>18<br><br>Saline-WM<br>7<br><br>Ketamine-Perception<br>4	Kruskal-Wallis one-way analysis of variance with post hoc testing	**Ketamine-WM**<br>**Cue:**<br>Pre-Injection, *mean*=1382.60, *median*=1388.31<br>Early Post-Injection, *mean*=1434.18, *median*=1439.26<br>Late Post-Injection, *mean*=1412.15, *median*=1411.39<br><br>*H*=14.16, *p*=0.0008, *df*=2,51<br>Post Hoc<br>Pre-Injection, Early Post-Injection, *p*=0.0005<br>Pre-Injection, Late Post-Injection, *p*=0.180<br>Early Post-Injection, Late Post-Injection, *p*=0.114

			**Delay:**<br>Pre-Injection, *mean*=866.65, *median*=890.28<br>Early Post-Injection, *mean*=939.50, *median*=946.17<br>Late Post-Injection, *mean*=901.11, *median*=938.40<br><br>*H*=11.15, *p*=0.004, *df*=2,51<br>Post Hoc<br>Pre-Injection, Early Post-Injection, *p*=0.003<br>Pre-Injection, Late Post-Injection, *p*=0.176<br>Early Post-Injection, Late Post-Injection, *p*=0.264<br><br>**Saline-WM**<br>**Cue:**<br>Pre-Injection, *mean*=1399.29, *median*=1407.69<br>Early Post-Injection, *mean*=1386.25, *median*=1377.65<br>Late Post-Injection, *mean*=1383.77, *median*=1380.16<br><br>*H*=0.27, *p*=0.872, *df*=2,18<br><br>**Delay:**<br>Pre-Injection, *mean*=875.11, *median*=863.25<br>Early Post-Injection, *mean*=873.23, *median*=839.04<br>Late Post-Injection, *mean*=890.06, *median*=892.58<br><br>*H*=0.14, *p*=0.932, *df*=2,18<br><br>**Ketamine-Perception**<br>**Cue:**<br>Pre-Injection, *mean*=1397.99, *median*=1397.98<br>Early Post-Injection, *mean*=1448.03, *median*=1447.44<br>Late Post-Injection, *mean*=1436.54, *median*=1443.50<br><br>*H*=3.85, *p*=0.146, *df*=2,9<br><br>**Delay:**<br>Pre-Injection, *mean*=879.42, *median*=896.63<br>Early Post-Injection, *mean*=954.42, *median*=953.70<br>Late Post-Injection, *mean*=942.55, *median*=945.68<br><br>*H*=1.5, *p*=0.472, *df*=2,9

**Extended Data 10g-l**<br>Percent of fixations on target location compared between injection periods	Ketamine-WM<br>18<br>*n*=9 target locations<br><br>Saline-WM<br>7<br>*n*=9 target locations<br><br>Ketamine-Perception<br>4<br>*n*=9 target locations	Kruskal-Wallis one-way analysis of variance	**Ketamine-WM**<br>**Cue:**<br>Pre-Injection, *mean*=22.5, *median*=24.2<br>Early Post-Injection, *mean*=26.9, *median*=24.6<br>Late Post-Injection, *mean*=24.0, *median*=24.9<br><br>*H*=0.84, *p*=0.658, *df*=2,24<br><br>**Delay:**<br>Pre-Injection, *mean*=26.9, *median*=26.2<br>Early Post-Injection, *mean*=24.9, *median*=20.8<br>Late Post-Injection, *mean*=28.2, *median*=26.5<br><br>*H*=1.03, *p*=0.598, *df*=2,24<br><br>**Saline-WM**<br>**Cue:**<br>Pre-Injection, *mean*=21.5, *median*=22.9<br>Early Post-Injection, *mean*=20.0, *median*=21.1<br>Late Post-Injection, *mean*=23.0, *median*=25.2<br><br>*H*=0.79, *p*=0.673, *df*=2,24<br><br>**Delay:**<br>Pre-Injection, *mean*=22.0, *median*=22.5<br>Early Post-Injection, *mean*=27.2, *median*=27.6<br>Late Post-Injection, *mean*=35.4, *median*=36.1<br><br>*H*=3.65, *p*=0.161, *df*=2,24<br><br>**Ketamine-Perception**<br>**Cue:**<br>Pre-Injection, *mean*=28.9, *median*=30.0<br>Early Post-Injection, *mean*=22.6, *median*=21.7<br>Late Post-Injection, *mean*=23.1, *median*=26.9<br><br>*H*=1.03, *p*=0.599, *df*=2,24<br><br>**Delay:**<br>Pre-Injection, *mean*=21.7, *median*=17.3<br>Early Post-Injection, *mean*=21.9, *median*=22.0

			Late Post-Injection, *mean*=20.6, *median*=20.3<br><br>*H*=0.7, *p*=0.704, *df*=2,24
